# Deciphering the immunomodulatory mechanisms of *Periplaneta americana L.* extract CII-3: insight from integrated metabolomics and network pharmacology

**DOI:** 10.3389/fcell.2025.1718560

**Published:** 2026-01-07

**Authors:** Yilin Wang, Yingxiang Wu, Xi Liu, Zhiyan Lu, Tianqian Li, Yang Jin, Yan Wang, Jiali Zhu

**Affiliations:** 1 Yunnan Provincial Key Laboratory of Entomological Biopharmaceutical R&D, Dali University, Dali, China; 2 National-Local Joint Engineering Research Center of Entomoceutics, Dali University, Dali, China; 3 School of Pharmacy, Dali University, Dali, China; 4 Department of Pharmacy, Traditional Chinese Medicine Hospital of Dali, Dali, China

**Keywords:** chemical components identification, immunoenhancing effects, metabolomics, network pharmacology, *Periplaneta americana L*, *.*

## Abstract

**Background:**

*Periplaneta americana* L*.* is one of the most famous traditional Chinese medicines (TCMs)*,* and CII-3 is the major bioactive extract of *Periplaneta americana* L. In recent years, CII-3 has gradually attracted the attention of researchers for its powerful capacity for treating immunocompromised diseases. However, systematical chemical composition investigation and mechanisms on immunomodulation of CII-3 have not been thoroughly scrutinized.

**Methods:**

The chemical ingredients of CII-3 were determined by ultra-performance liquid chromatography tandem quadrupole time-of-flight mass spectrometry (UPLC-Q-TOF/MS), and immune-enhancing mechanisms and active constituents were investigated by integration of metabolomics with network pharmacology and molecular docking.

**Results:**

A total of 25 components were identified in the aqueous extract of CII-3, among which 10 were unambiguously confirmed by comparison with reference standards. In immunosuppressed rats, oral administration of CII-3 normalized biochemical profiles by promoting the secretion of immune-related cytokines (IL-2, IL-6) and immunoglobulins (IgG, IgM), significantly improving immune organ indices, and alleviating pathological lesions in the thymus and spleen. Furthermore, CII-3 stimulation attenuated CTX-induced upregulation of IL-6 mRNA levels and concurrently enhanced IL-2 mRNA expression in these two immune organs. Based on metabolomics analysis, 27 differential metabolites along with 7 crucial metabolic pathways including starch and sucrose metabolism, arginine biosynthesis, porphyrin and chlorophyll metabolism, nicotinate and nicotinamide metabolism, steroid biosynthesis, pyrimidine metabolism and tryptophan metabolism were regulated after CII-3 treatment, suggesting that CII-3 has the potential to target these metabolites and key pathways to improve immunosuppression. Nitric oxide synthase 1 (NOS1), nitric oxide synthase 3 (NOS3), acetylcholinesterase (ACHE), cluster of differentiation 38 (CD38) and poly (ADP-ribose) polymerase 1 (PARP1) were considered as the pivotal targets, and ginsenine, cyclo (Tyr-Asp), guanosine, tryptophan and inosine were deemed potential active components of CII-3 in alleviating immunosuppression.

**Conclusion:**

The proposed approach provides a valuable evidence for demonstrating the material basis of CII-3. The results obtained in the present study elucidated that the potential immunomodulatory activity of CII-3 might be closely associated with these crucial targets, which can modulate the levels of major metabolites through multiple metabolic pathways, thus ameliorating immunosuppression.

## 1 Introduction

Immune system is an important defense system against foreign invasion. Immunosuppression is considered a significant threat to human health worldwide. Many factors can cause immunosuppression, including genetics, age, irregular work and rest habits, stress, and immunosuppressive agents (Chen et al., 2021; Tran et al., 2023), leading to the occurrence of various diseases. Millions of people worldwide nowadays have suffered from immunosuppressive diseases. Conditions such as Type 1 Diabetes Mellitus, Psoriasis, and Rheumatoid Arthritis currently affect approximately 10% of the global population, with prevalence expected to rise further in the coming years. Despite advancements in medical science and improved access to healthcare, the rising incidence and mortality of these diseases continue to present significant public health challenges, imposing substantial burdens on both patients’ physical and mental wellbeing, as well as on healthcare systems worldwide. Therefore, immunopotentiation therapy has long been used in medicine to improve host defense and disease resistance. However, the long-term use of immunomodulators can cause severe adverse reactions, such as anemia, stomach pain, nerve damage, abdominal pain, and even abnormal damage to the liver (Tran et al., 2023; Chu et al., 2020). Therefore, finding drugs with significant effects and minimal side effects to treat immunosuppression are particularly critical.

TCMs have been used in China for millennia (Gu et al., 2022; Huang et al., 2025). Among them, medicinal insects have been shown to modulate immune responses, such as cinobufagin and deer velvet (Yu et al., 2022; Baek et al., 2023). *Periplaneta americana* L. is a classic TCM, and its medicinal application was firstly recorded in “*Shen Nong’s Herbal Classics*” (Xu et al., 2022). It has been documented in the medicinal material standards of Yunnan, Sichuan, Hunan and other provinces (Ma et al., 2022). In recent years, the multiple pharmacological activities of *Periplaneta americana* L. has gradually attracted the attention of researchers and shown good application prospects for its anti-tumor, anti-inflammatory, anti-hepatocellular carcinoma, anti-gastric ulcer, tissue repair and immune-enhancing effects (Ma et al., 2022; Zhao et al., 2017; Nguyen et al., 2020; Zhang et al., 2022; Wang et al., 2024). Yang et al. (Yang et al., 2020) reported CII-3, the main bioactive extract of *Periplaneta americana* L., has an immunoprotective effect on Lewis tumor-bearing mice. Besides, modern research demonstrated that CII-3 contains physiologically active components such as amino acids (Xu et al., 2022; Wang et al., 2024), nucleosides (Xu et al., 2022; Wang et al., 2024), peptides (Wang et al., 2023) and proteins (Wu et al., 2023). Previous studies have reported that CII-3 could inhibit the polarization of M2 macrophages (Xu et al., 2022), and enhance the effect of immunity (Xu et al., 2022; Yang et al., 2020). These studies have solely focused on pharmacodynamic evaluation of CII-3 but few scientific reports on its compositions and mechanism of action. JI et al. (Ji et al., 2021) has demonstrated that CII-3 can achieve anti-tumor effect might be related to enhance immune function. However, the pharmacological mechanism-related research in terms of immune-enhancing effects of CII-3 remains unclear until now. Therefore, the present study aimed to clarify the material basis of CII-3 by means of UPLC-Q-TOF/MS, to decipher the potential protective mechanisms of CII-3 against CTX-induced immunosuppression by integration of metabolomics with network pharmacology and molecular docking. The investigations present a panorama of molecular mechanisms of CII-3, which not only substantiates the immunomodulatory effect of this extract, but also sheds some light on the immunosuppressive therapy of *Periplaneta americana* L.

## 2 Methods

### 2.1 Analysis of the chemical compounds of CII-3

#### 2.1.1 Preparation of sample solution

Freeze-dried powder of CII-3 (1.0 g) was accurately weighed and then extracted with 10 mL deionized water for 10 min ultrasound treatment. The aqueous extraction was filtered with a 0.22 μm microporous membrane to obtain the solution for the detection of CII-3 components.

#### 2.1.2 Preparation of reference solution

Each reference compound (lysine, arginine, tyrosine, tryptophan, phenylalanine, hypoxanthine, xanthine, adenine, inosine, and guanosine) was separately weighed and dissolved in methanol/water (1:1, v/v) to prepare individual stock solutions at 1.0 mg/mL. A mixed reference solution (with each analyte at approximately 200 ng/mL) was then prepared via two-step serial dilution: first, 20.0 μL of each 1.0 mg/mL individual stock solution was mixed and diluted to 10 mL with methanol/water (1:1, v/v) to obtain a 20 μg/mL intermediate mixed stock solution; second, 100.0 μL of the intermediate solution was further diluted to 10 mL with the same solvent. All the solutions were kept at −20 °C before qualitative analysis.

#### 2.1.3 UPLC-Q-TOF/MS conditions

ZORBAX SB-Aq (4.6 × 150 mm, 5 μm, Agilent, Milford, MA, USA) was employed for chromatographic separation of CII-3 on an Agilent 1290 UPLC system. A mobile phase consisting of acetonitrile (A) and 0.1% formic acid in water (B) was performed with the following gradient elution program: 100% B at 0 min, 100%–99.5% B at 0–1 min, 99.5%–99.0% B at 1–3 min, 99.0%–96.5% B at 3–11.5 min, 96.5%–90.0% B at 11.5–15 min, 90.0% B at 15–16 min. The column temperature and the flow rate were maintained at 30 °C and 1.0 mL/min. The injection volume was 5 µL. An Agilent 6540 UHD Q-TOF mass spectrometry (Agilent, Milford, MA, USA), equipped with an electrospray ionization (ESI) source was conducted for mass spectrometric detection in both positive and negative ionization mode with scan range of *m/z* 100–1700 Da for MS and *m/z* 50–1700 Da for MS/MS. The flow rate of desolvation gas (Ar) was 8 L/min at a temperature of 350 °C. Capillary voltage was maintained at 3500 V in both ESI^+^ and ESI^−^, and the collision energies were set at 10, 20, 30, 40 and 50 eV, respectively. Mass was corrected using leucine-enkephalin, which monitored a reference ion at *m/z* 556.2776 [M + H]^+^ for ESI^+^ and at *m/z* 554.2620 [M-H]^-^ for ESI^−^.

### 2.2 Network pharmacology of CII-3 against immunosuppression

Candidate targets of CII-3 compounds were predicted using databases of BATMAN (http://bionet.ncpsb.org.cn/batman-tcm/), DrugBank (https://www.drugbank.ca/) and Swiss Target Prediction (http://www.swisstargetprediction.ch/). The keywords of “immune regulation”, “immunocompromised”, “immunomodulation” and “immunosuppression” were searched using databases of GeneCards (https://www.genecards.org/), OMIM (https://www.omim.org/), PharmGKB (http://www.pharmgkb.org/), and DisGeNET (http://www.disgenet.org/) to identify candidate targets for related diseases. Integration of molecular targets and disease targets after de-duplication, the overlapped targets were considered as the potential targets for immunoregulation exerted by CII-3. Protein-protein interaction (PPI) network was generated using STRING (https://cn.string-db.org/) and Cytoscape (https://cytoscape.org/). Gene ontology (GO) and Kyoto Encyclopedia of Genes and Genomes (KEGG) enrichment analyses of potential targets were conducted using the “clusterProfiler” R software (Version 4.4.2). A significance threshold of *P*-value ≤0.05 and Q-score ≤0.05 was applied to identify statistically meaningful results. The top 10 GO terms and the top 10 KEGG pathways were displayed.

### 2.3 Animals experiment

A total of 36 healthy male Sprague-Dawley (SD) rats (SPF, weight 180–200 g, aged 6–8 weeks) were purchased from Spef Biotechnology Co., Ltd. (Beijing, China, Permit number: SCXK (jing) 2019-0010). The rats were housed at a temperature of 25 °C ± 2 °C and a relative humidity of 60% ± 10% under a 12 h light-dark cycle, and received food and water *ad libitum*.

All of the animal experimental procedures were approved by the Ethics Committee for Animal Care and Use of Dali University (approval number: 2021-P2-033), and were performed in strict according to the guidance for the Administration of Affairs Concerning Experimental Animals of China. To ensure animal welfare, measures such as ventilation and regular bedding changes were implemented to achieve environmental enrichment. Throughout the experiment, the animal welfare was conducted in accordance with the Guideline for Ethical Review of Laboratory Animal Welfare ensuring that all the procedures were humane and cause minimal pain to laboratory animal.

After 1 week of acclimatization, 36 rats were randomly assigned into 6 groups (six rats per group) as follows: the control group, the model group (CTX group), the positive control group (LM group), CII-3 with 25 mg/kg, 50 mg/kg and 100 mg/kg respectively in the low, medium and high dose of CII-3 groups (the doses of CII-3 in rats were selected based on preliminary experiments). Except for the control group, all rats were received intraperitoneal injection of CTX (40 mg/kg) once a day for days 1, 2, 3, 6 and 10. CII-3 groups were orally administered different dosage of CII-3 once daily for 14 consecutive days. Rats of positive control group were orally treated with LM (50 mg/kg) on the same schedule. An equal volume of distilled water was orally administered to the control and CTX groups. The body weight of rats were recorded every 3 days during the experimental period. After 24 h of last administration, isoflurane was used to anesthetize all animals. Blood samples were collected from the abdominal aorta, left at room temperature for 1 h and then centrifuged at 4000 r/min at 4 °C for 10 min, to obtain serum samples. The serum was then frozen in liquid nitrogen and stored at −80 °C. Spleen and thymus were instantly isolated and immediately washed with ice-cold physiological saline, and then recorded the weights. All samples were stored at −20 °C before use.

### 2.4 Animal model assessment

#### 2.4.1 Assessments of immune organ indices

The spleen and thymus indices were calculated according to the following formula: organ index = spleen or thymus weight (g)/body weight (g)*100%.

#### 2.4.2 Determination of immune cell levels

The amount of white blood cells (WBC), red blood cells (RBC) and blood platelet (PLT) of all rat samples were determined using fully automated hematology analyzer.

#### 2.4.3 Determination of serum cytokine and immunoglobulin levels

The contents of cytokine (IL-2 and IL-6) and immunoglobulin (IgG and IgM) in the serum were detected using ELISA kits according to the manufacturer’s instructions.

#### 2.4.4 Quantitative real-time reverse transcription-polymerase chain reaction (qRT-PCR)

30–50 mg of rat spleen and thymic tissues were collected and ground into powder in liquid nitrogen. Total RNA was extracted using a total RNA isolation kit, and the concentration and purity were determined with a Nano Drop 2000 spectrophotometer. Reverse transcription of the purified RNA into cDNA was performed using the fastking RT kit (with gDNase). qRT-PCR was carried out on a PCR system with superreal premix plus (SYBR Green), and the specificity of PCR amplification was validated by melting curve analysis. Quantitative detection was conducted utilizing the 2^(−ΔΔCt)^ method. To reduce experimental variability, each sample was analyzed in three technical replicates. β-actin was selected as the internal reference gene for calculating the relative expression levels of target genes, and all primer sequences were provided in Supplementary Table S1.

#### 2.4.5 Histopathological examinations of spleen and thymus

A certain amount of spleen and thymus of individual rat were respectively cut and stored in formalin, and then dehydrated with ethanol and embedded in paraffin wax. Finally, tissue sections were prepared with hematoxylin and eosin (HE) staining, observed under the light microscope and photographed.

### 2.5 Metabonomics analysis

#### 2.5.1 Sample pretreatment and the LC-MS/MS conditions

All of the serum samples were stored at −20 °C and allowed to thaw at room temperature before processing. 400 μL of methanol containing 4 ppm 2-chloro-L-phenylalanine (internal standard) was added to 100 μL of serum, and then the mixture was vortexed thoroughly for 1 min and centrifuged at 13,000 rpm at 4 °C for 10 min, the supernatant was collected and evaporated to dryness under a gentle stream of nitrogen. The residue was reconstituted with 150 μL of methanol/water (4:1, v/v), vortexed for 3 min, and centrifuged at 13,000 rpm for 10 min. Then, the supernatant was collected for analysis.

LC analysis was implemented on an ACQUITY UPLC system (Waters Corporation, Milford, MA, USA). The chromatographic separation of analytes was performed on an ACQUITY UPLC® HSS T3 (2.1 × 150 mm, 1.8 µm) column at 40 °C. The mobile phase was composed of water containing 0.1% formic acid (A, v/v) and acetonitrile containing 0.1% formic acid (B, v/v) with flow rate of 0.25 mL/min. The LC gradient elution condition was listed as follows: 0–1 min, 98% A; 1–9 min, 98%-50% A; 9–12 min, 50%–2% A; 12–13.5 min, 2% A; 13.5–14 min, 2%–98% A; 14–20 min, 98% A. The injection volume was maintained at 2 μL.

Mass spectrometry was performed using a Thermo Q-Exactive mass spectrometer which equipped with an ESI operating in positive and negative ion modes. The ion spray voltage was set at 3.5 KV (ESI^+^) and −2.5 KV (ESI^−^), and the capillary temperature was 325 °C. The scanning range was 100–1,000 *m/z* for full scan at a mass resolution of 70,000, MS/MS experiment was executed using a high-energy collisional dissociation scan with collision energy of 30 eV.

#### 2.5.2 Method validation

Quality control (QC) samples were prepared by pooling each samples (control group, model group and CII-3M group) with the same volume. QC samples were injected among groups during the entire detection to monitor the system stability in both ESI^+^ and ESI^−^. The *m/z* values of ten peaks in QC samples were selected in ESI^+^ and ESI^−^, and the relative standard deviations (RSDs) of the retention times and peak areas were calculated.

### 2.6 Molecular docking analysis

The 3D protein structures were downloaded from the RCSB PDB database (https://www.rcsb.org/), after which water molecules and modified ligands were removed using PyMOL software (Version 2.6.0a0). The 2D structures of compounds obtained from PubChem database (https://pubchem.ncbi.nlm.nih.gov/) underwent energy minimization in Chem3D software (Version 20.0). The optimized compounds were then subjected to molecular docking with the proteins using AutoDock Vina software (Version 1.1.2), followed by visualization of docking results through PyMOL.

### 2.7 Data analysis

All of the experimental data were represented as mean ± standard deviation. All statistical analyses were performed using SPSS (Statistical Product and Service Solutions Inc., Chicago, IL, USA) 19.0 Statistical software. The Shapiro-Wilk test was used to assess the normality of continuous variables. For variables that fulfilled the normality assumption, one-way analysis of variance (ANOVA) was performed to analyze differences among multiple groups. Homogeneity of variances was evaluated using the Levene test. If the assumption of homogeneity was met (P ≥ 0.05), post-hoc multiple comparisons were performed using the least significant difference test. Conversely, if the assumption was violated (P < 0.05), Dunnett’s T3 method was applied for multiple comparisons. A two-sided P-value of <0.05 was considered statistically significant. All statistical figures were depicted utilizing GraphPad Prism (GraphPad Software, Inc., La Jolla, CA, USA) 7.0 software.

Data processing for components identification of CII-3 were performed using Masshunter software (version B.06.00). The metabolites were confirmed by accuracy relative molecular mass (<10 ppm) and MS/MS fragmentations were compared with databases of massbank (https://massbank.eu/), KEGG (https://massbank.eu/), lipidmaps (https://www.lipidmaps.org/), mzcloud (https://www.mzcloud.org/) and HMDB (https://hmdb.ca/).

After normalization, metabolites presenting in at least 80% of all the samples were kept for further analysis, and only peaks with RSDs of peak area less than 30% in QC samples were selected to ensure appropriate metabolites identification. Multivariate statistical analysis was conducted using the SIMCA-P (Umetrics AB, Umea, Sweden) 14.1 software. The qualities of partial least-square discriminant analysis (PLS-DA) and orthogonal partial least-square discriminant analysis (OPLS-DA) models were described by R2X, R2Y, and Q2 and the models were evaluated for over-fitting with methods of permutation tests (with 200 permutations). Metabolites with *P* value <0.05 and variable importance projection (VIP) value >1 yielded by OPLS-DA were identified to be differential metabolites (biomarkers). The most relevant metabolic pathways (an impact value >0.10) of potential biomarkers were analyzed using MetaboAnalyst 5.0 (https://www.metaboanalyst.ca/).

## 3 Results

### 3.1 Identification of chemical constituents of CII-3

**FIGURE 1:**
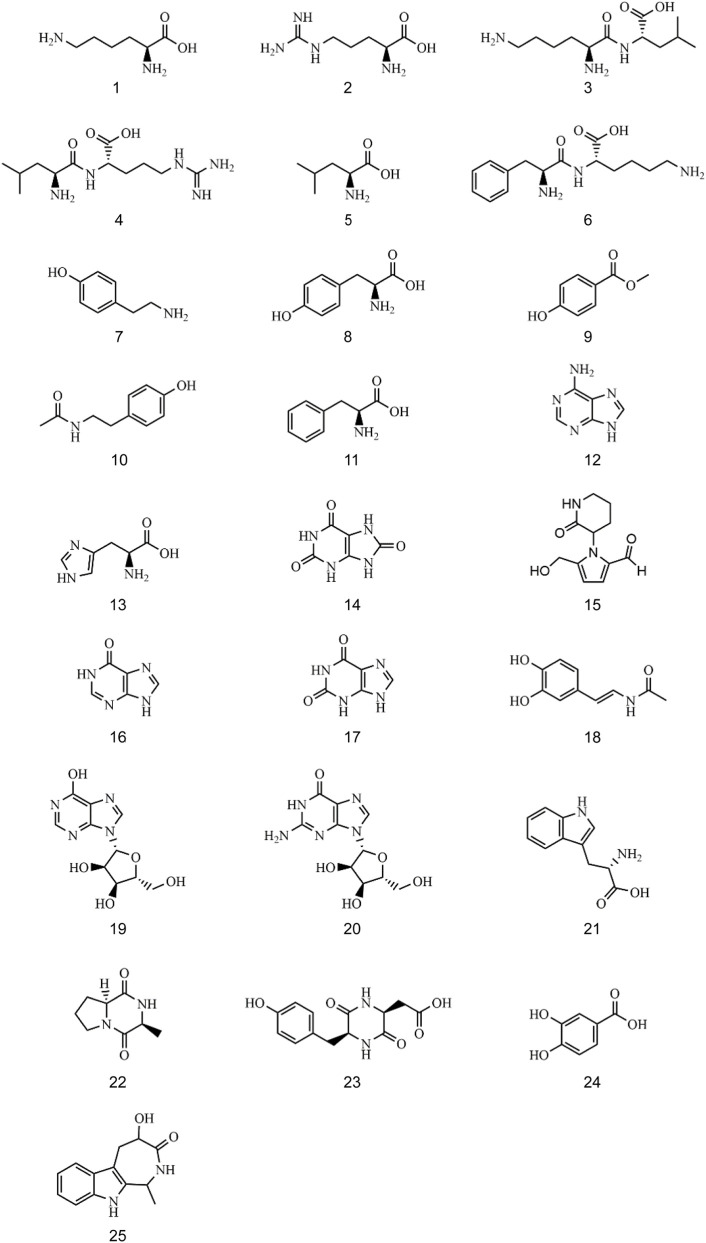
The chemical structures of twenty-five ingredients in CII-3.

The mixed reference solution and sample solution of CII-3 were analyzed under the optimal conditions, and the base peak ion chromatograms (BPC) obtained in ESI^+^ and ESI^−^ by UPLC-Q-TOF/MS were shown in Supplementary Figure S1. Twenty-five constituents were characterized from the aqueous extract of CII-3, including seven amino acids, five dipeptides and cyclic peptides, three purines, two nucleosides, and eight others (Supplementary Table S2). Amongst them, ten compounds were unambiguously identified by comparison with the reference substances, and other compounds were identified after searching databases and referring to related literatures (Zhou et al., 2020; Calderón-Santiago et al., 2012; Liu et al., 2019; Vishwanathan et al., 2000; Chae et al., 2011; Dong et al., 2018; Prinsen et al., 2016; Servillo et al., 2014; Sagratini et al., 2012; Zhang et al., 2019; Sanchis et al., 2019; Heidari and Mohammadipanah, 2018; Furey et al., 2005; Wilkerling et al., 2012; Lee et al., 2006; Rukdee et al., 2015; Zhang and Xu, 2014; Xiang et al., 2018; Rashed et al., 2005; Stentoft et al., 2014; Meng et al., 2019; Xiao et al., 2007; Wang et al., 2016; Wu et al., 2007). The relative molecular mass was detected within a reasonable degree of accuracy (<10 ppm). The chemical structures of the twenty-five ingredients of CII-3 characterized by UPLC-Q-TOF/MS were listed in Figure 1.

#### 3.1.1 Identification of amino acid compounds

**FIGURE 2:**
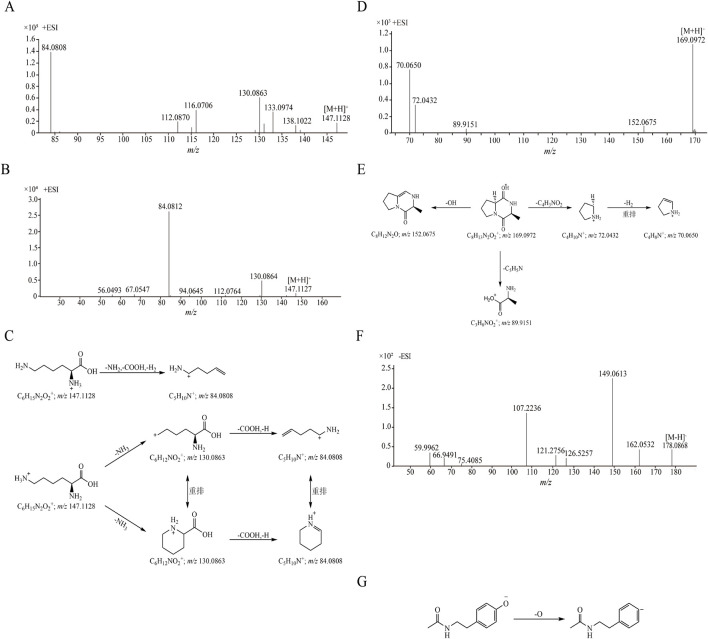
The MS/MS spectra and proposed fragmentation pathways of compounds. **(A)** The MS/MS spectrum of compound 1 in CII-3; **(B)** The MS/MS spectrum of reference substance lysine; **(C)** The proposed fragmentation pathways of lysine; **(D)** The MS/MS spectrum of compound 22 in CII-3; **(E)** The proposed fragmentation pathways of cyclo (Ala-Pro); **(F)** The MS/MS spectrum of compound 10 in CII-3; **(G)** The proposed fragmentation pathways of N-acetyltyramine.

The retention time of compound 1 was 1.361 min, and its quasi-molecular ion peak was *m/z* 147.1128 [M + H]^+^ in ESI^+^ and *m/z* 145.0977 [M-H]^-^ in ESI^−^, respectively. Its calculated molecular formula was predicted to be C_6_H_14_N_2_O_2_. In the MS/MS spectrum (Figure 2A), one molecule of NH_3_ was easily lost from the precursor ion [M + H]^+^ to form a fragment ion at *m/z* 130.0863 [M + H-NH_3_]^+^, the ion at *m/z* 130.0863 could further lose one molecule of carboxyl group and a hydrogen atom to produce ion at *m/z* 84.0808 [M + H-NH_3_-COOH-H]^+^. Under the same conditions, the retention time of the reference substance lysine was 1.359 min, the quasi-molecular ion was *m/z* 147.1127 [M + H]^+^ and the fragment ions were *m/z* 130.0864 and *m/z* 84.0812 (Figure 2B). After comparison with the reference substance, compound 1 was unambiguously attributed to lysine. The proposed fragmentation pathways of lysine in ESI^+^ was shown in Figure 2C. Seven amino acid compounds (compositions 1, 2, 5, 8, 11, 13 and 21) were identified (details in Supplementary Figures S2–S7).

#### 3.1.2 Identification of dipeptide compounds

Compound 22 exhibited the [M + H]^+^ ion at *m/z* 169.0972 in positive ion mode in 11.001 min, its calculated molecular formula was predicted to be C_8_H_12_N_2_O_2_. The main fragment ions analyzed by MS/MS screening were observed at *m/z* 152.0675 [M + H-OH]^+^, *m/z* 89.9151 [M + H-C_5_H_5_N]^+^, *m/z* 72.0432 [M + H-C_4_H_3_NO_2_]^+^ and *m/z* 70.0650 [M + H-C_4_H_3_NO_2_-H_2_]^+^ in the positive ion spectrum (Figure 2D). After databases search (e.g., https://www.allpeptide.com/, https://www.chemicalbook.com/) and comparison its relative molecular mass and MS/MS spectrum with literature data (Zhou et al., 2020), compound 22 was deduced as a cyclo (Ala-Pro). The proposed fragmentation pathways of cyclo (Ala-Pro) in ESI^+^ was shown in Figure 2E. Five dipeptide and cyclic peptide compounds (compositions 3, 4, 6, 22 and 23) were determined (Supplementary Figures S8–S11).

#### 3.1.3 Identification of nucleoside compounds

**FIGURE 3:**
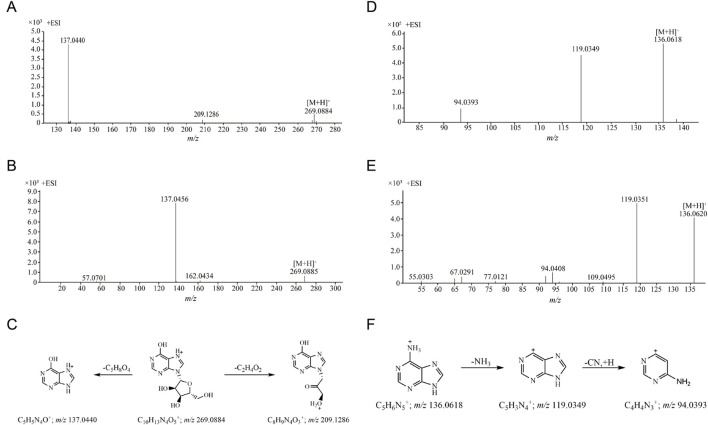
The MS/MS spectra and proposed fragmentation pathways of compounds. **(A)** The MS/MS spectrum of compound 19 in CII-3; **(B)** The MS/MS spectrum of reference substance inosine; **(C)** The proposed fragmentation pathways of inosine; **(D)** The MS/MS spectrum of compound 12 in CII-3; **(E)** The MS/MS spectrum of reference substance adenine; **(F)** The proposed fragmentation pathways of adenine.

The retention time of compound 19 was 8.331 min, and its quasi-molecular ion peak was *m/z* 269.0884 [M + H]^+^ in ESI^+^ and *m/z* 267.0720 [M-H]^-^ in ESI^−^, respectively. Its calculated molecular formula was predicted to be C_10_H_12_N_4_O_5_. The daughter ion at *m/z* 209.1286 suggested the loss of a molecular of C_2_H_4_O_2_ have been occurred. The *m/z* 137.0440 [M + H-C_5_H_8_O_4_]^+^ fragment ion appeared when one molecule of ribofuranose was lost in the MS/MS spectrum (Figure 3A). Under the same conditions, the retention time of the reference substance inosine was 8.327 min, the quasi-molecular ion was *m/z* 269.0885 [M + H]^+^ and the fragment ion peak *m/z* 137.0456 was formed (Figure 3B). After comparison with the reference substance, compound 19 was identified as inosine. The proposed fragmentation pathways of inosine in ESI^+^ was shown in Figure 3C. Two nucleoside compounds (compositions 19 and 20) were identified (Supplementary Figure S12).

#### 3.1.4 Identification of purine compounds

Compound 12 (*m/z* 136.0618 [M + H]^+^ in 4.675 min) was detected with detailed precursor-to-product ions shown in Figure 3D, some diagnostic fragment ions at *m/z* 119.0349 and *m/z* 94.0393 have been observed, suggesting that it was apt to undergoing the neutral loss of one NH_3_ (17 Da) and sequentially one CN (27 Da) moiety from the precursor ion [M + H]^+^. By comparing with the retention time and its accurate parent and daughter ions of reference compound (Figure 3E), compound 12 was confirmed as adenine. The proposed fragmentation pathways of adenine in ESI^+^ was shown in Figure 3F. Three purine compounds (compositions 12, 16 and 17) were identified (Supplementary Figures S13, S14).

#### 3.1.5 Identification of other compounds

The retention time of compound 10 was 3.909 min. Under ESI^−^, its quasi-molecular ion peak was *m/z* 178.0868 [M-H]^-^, and its calculated molecular formula was predicted to be C_10_H_13_NO_2_. In the MS/MS spectrum (Figure 2F), it easily lost a oxygen atom to form the fragment ion *m/z* 162.0532 [M-H-O]^-^, additionally, the fragment ion *m/z* 149.0613 [M-H-CHO]^-^ was produced when one molecule of formyl group was lost. By comparing with the literature data (Heidari and Mohammadipanah, 2018), compound 10 was deduced as N-acetyltyramine. The proposed fragmentation pathways of N-acetyltyramine in ESI^−^ was shown in Figure 2G. Eight other compounds (compositions 7, 9, 10, 14, 15, 18, 24 and 25) were identified (Supplementary Figures S15–S21).

### 3.2 Network pharmacological analysis of CII-3 against immunosuppression

**FIGURE 4:**
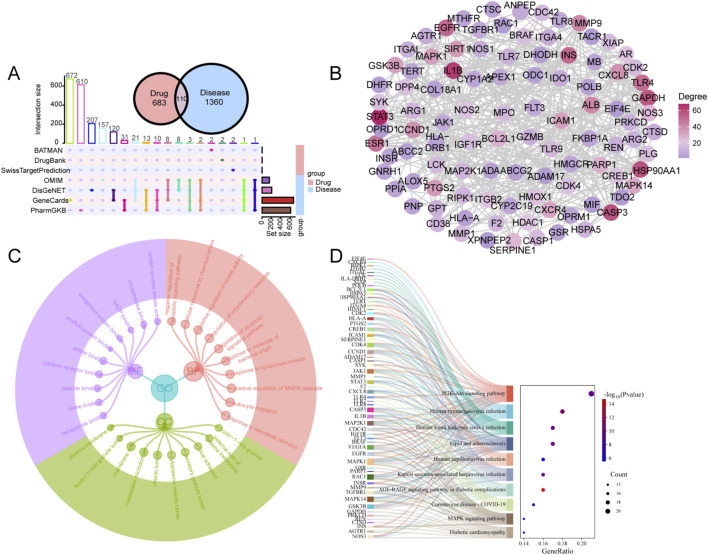
Network pharmacological analysis of CII-3 for the treatment of immunosuppression. **(A)** Upset plot of the intersectional targets between CII-3 compounds and disease. **(B)** PPI network of intersectional targets. Each node represents a gene and each edge represents an interaction between two genes. The nodes are color-coded according to their degree, warmer colors (red and orange) represents higher degrees (more connections) and cooler colors (green and purple) represents lower degrees (fewer connections). **(C)** GO enrichment analysis. The top 10 GO terms including BP, CC and MF by count were listed. **(D)** Top 10 pathways of KEGG enrichment analysis.

A total of 793 targets of CII-3 components and 1,470 relevant disease targets were obtained. There were 110 targets involved in both CII-3 and disease (Figure 4A). The 110 intersectional targets were imported into STRING for a PPI analysis. A minimum required interaction score of 0.7 was applied. After removing disconnected nodes, the PPI network consisted of 101 nodes and 888 edges (Figure 4B). The GO enrichment analysis incorporated three classical subschemas, i.e., biological process (BP), cellular component (CC), and molecular function (MF). The BP was mainly involved in response to xenobiotic stimulus, positive regulation of MAPK cascade, response to lipopolysaccharide and regulation of inflammatory response (Figure 4C). The CC was distributed in plasma membrane raft, membrane microdomain and cytoplasmic vesicle lumen. The MF mainly involved in protein tyrosine kinase activity, phosphatase binding and cytokine receptor binding. KEGG pathway analysis indicated 156 pathways were enriched, and the top 10 pathways, ranked by *P*-value, related to PI3K-Akt, lipid and atherosclerosis, AGE-RAGE, and MAPK signaling pathway (Figure 4D).

### 3.3 Activity against immunosuppression for CII-3

#### 3.3.1 CII-3 increases the levels of immune cells in the blood of CTX-induced rats

**FIGURE 5:**
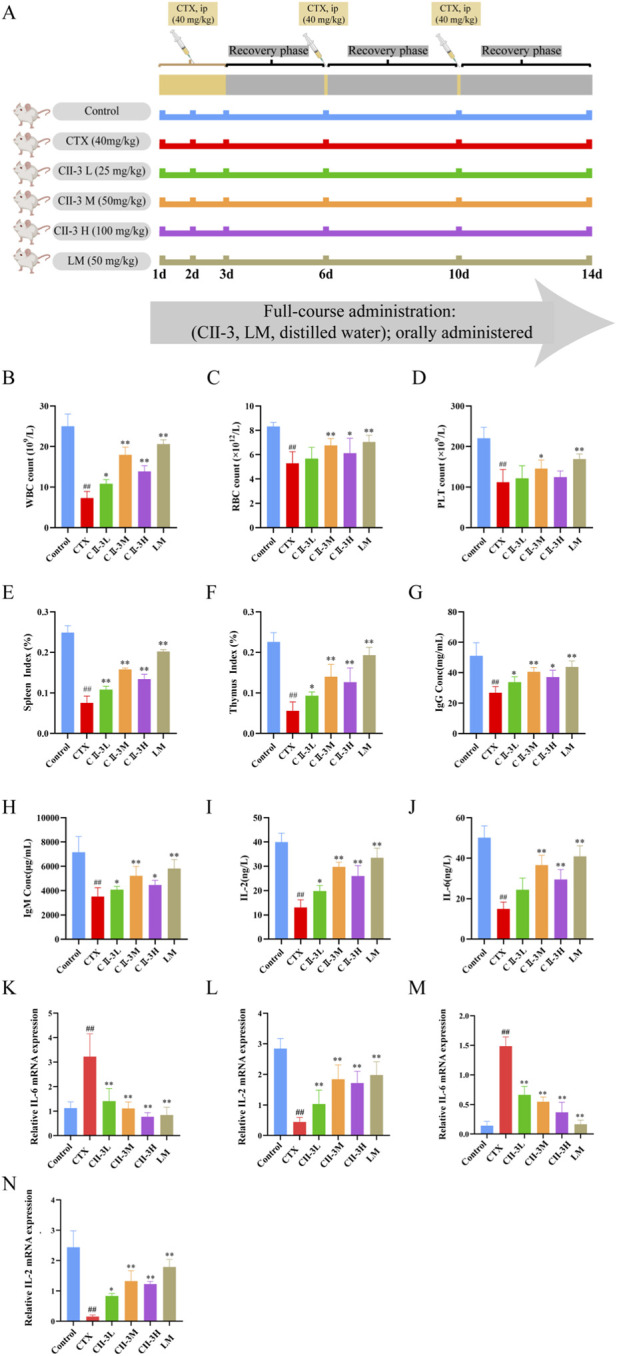
Pharmacodynamic evaluation of CII-3 against immunosuppression. **(A)** Experimental design of CII-3 treatment of CTX-induced immunosuppression in SD rats; **(B–D)** Effect of CII-3 on WBC, RBC and PLT counts. **(E,F)** Effect of CII-3 on spleen and thymus indices in CTX-induced immunosuppressive rat. **(G–J)** Effect of CII-3 on the serum levels of immunoglobulins and cytokines. **(K)** IL-6 mRNA expression in thymus tissue of rats. **(L)** IL-2 mRNA expression in thymus tissue of rats. **(M)** IL-6 mRNA expression in spleen tissue of rats. **(N)** IL-2 mRNA expression in spleen tissue of rats. Data are expressed as mean ± SD. ^##^ *P* < 0.01 compared with the control group; ^*^ *P* < 0.05, ^**^ *P* < 0.01 compared with the model group.

Figure 5A displays the schematic diagram of the animal experiment. Since the amount of WBC reflects the body’s humoral immunity, blood routine analysis elucidated that CTX significantly (*P* < 0.01) decreased the amount of WBC, RBC and PLT (Figures 5B–D), indicating the success of the immunosuppressive modeling in this study. Compared with CTX group, CII-3 could significantly (*P* < 0.05) increase the WBC, RBC and PLT counts in peripheral blood of rat, thus alleviating the immunosuppression caused by CTX, and especially the CII-3M group had greater levels of RBC (*P* < 0.01) and PLT (*P* < 0.05) than the CTX group.

#### 3.3.2 CII-3 ameliorates spleen and thymus indices induced by CTX

Compared to the control group, the spleen and thymus indices decreased (*P* < 0.01) after exposure to CTX (Figures 5E,F), these results manifested that the immune function of rats in CTX group was disturbed, resulting in immunosuppression. The spleen and thymus indices were substantially restored when rats were treated with CII-3 (*P* < 0.05). The spleen index of rat in all dose groups of CII-3 was significantly (*P* < 0.01) higher than that in CTX group (Figure 5E), while the thymus index of rat in CII-3M and CII-3H groups was significantly (*P* < 0.01) higher than that in CTX group (Figure 5F). The results demonstrated that CII-3 could reverse the atrophy of immune organs caused by CTX.

#### 3.3.3 CII-3 alleviates the CTX-induced decrease of serum cytokine and immunoglobulin levels

As shown in Figures 5G–J, IgG, IgM, IL-2 and IL-6 secretions in CTX-treated rats were dramatically decreased (*P* < 0.01) compared to those in the control group, indicating that CTX successfully induced immunosuppression. Conversely, significant improvements in serum immunoglobulin and cytokine levels were observed in rats treated with CII-3 (*P* < 0.05) compared to CTX group. Especially, the CII-3M treatment group (the oral administration of CII-3 at a dose of 50 mg/kg) increased the expression levels of IgG, IgM, IL-2 and IL-6 as effectively as the LM treatment group. Studies demonstrated that CII-3 showed strong restoration of immune-related factors, and it has the potential to increase adaptive immunity in immunosuppressed rat by reducing the expression of inflammatory factors.

#### 3.3.4 CII-3 reverses the CTX-induced elevation of IL-6 mRNA level and reduction in IL-2 mRNA level in the spleen and thymus

Compared with the control group, IL-6 mRNA expression was significantly upregulated (*P* < 0.01) in the model group. In contrast, in the spleen and thymus, IL-6 mRNA expression was significantly downregulated (*P* < 0.01) in the CII-3L, CII-3M, and CII-3H groups compared with the model group (Figures 5K,M).

As shown in Figures 5L,N, compared with control group, the mRNA expression of IL-2 rats in the model group was extremely low (*P* < 0.01). Compared with the model group, the mRNA expression of IL-2 rats in CII-3M and CII-3H group increased significantly (*P* < 0.01); Therefore, CII-3M and CII-3H significantly inhibited the IL-6 mRNA expression level and promoted the IL-2 mRNA expression level in rat spleen and thymus tissues (*P* < 0.01).

#### 3.3.5 Histopathological examination

**FIGURE 6:**
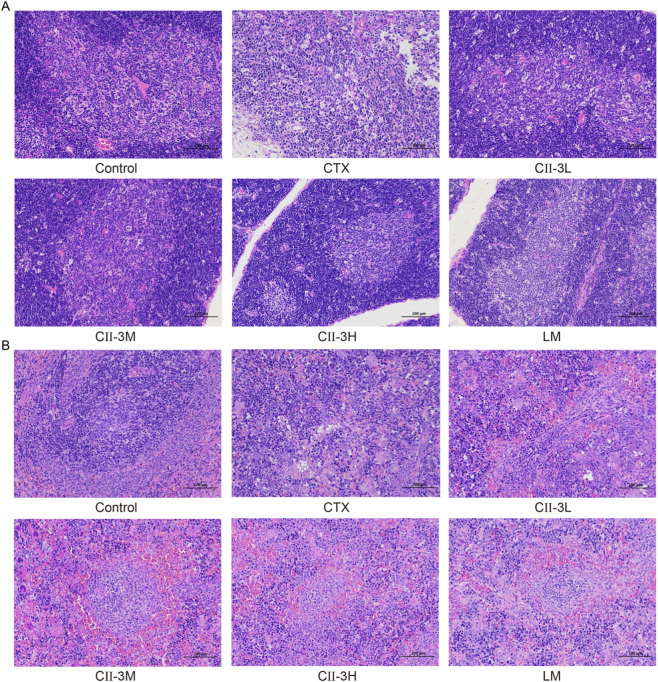
Effect of CII-3 on the histological morphology of thymus **(A)** and spleen **(B)** in CTX-induced rat. Scale bar: 100 μm.

Figure 6 displays the histological morphology of thymus and spleen of rats. As a central immune organ, the thymus plays a pivotal role in T lymphocyte differentiation and immunomodulation. In the control group (Figure 6A), the division between the cortex and medulla was distinct in the thymus, the cortex was dark, and the medulla was light. However, the defined areas between the cortex and medulla became obviously obscured following CTX treatment, which implied that the compartmentalization in the thymus degenerated. The damage to the thymus was relieved to varying degrees after CII-3 treatment compared to the CTX group.

The splenocytes in the control group were dense and well-arranged, what’s more, there were clear boundaries between white pulp and red pulp (Figure 6B). Nevertheless, in CTX group, the white pulp was destroyed and the structure was disordered. There was no obvious dividing line between white pulp and red pulp, and there were few lymphocytes. After the intervention of CII-3, the demarcation lines of red and white pulp became obvious, and the number of lymphocytes were substantially increased. These results suggested that CII-3 could effectively ameliorate the damage of CTX on thymus and spleen organs.

### 3.4 Metabonomics results

#### 3.4.1 Metabolomics profiling of the serum samples

Under the optimized LC-MS/MS conditions, BPC of the serum samples were analyzed in both ESI^+^ and ESI^−^ (Supplementary Figure S22). The endogenous components obtained satisfactory separation within 20 min.

#### 3.4.2 Method validation

As shown in Supplementary Figure S23, tight cluster of QC samples represented in the score matrix of principle component analysis (PCA) for both ESI^+^ and ESI^−^ assure instrumental robustness throughout the entire LC-MS/MS analysis procedure. Besides, 10 ions were randomly extracted from the BPC of QC samples for method validation. The RSDs of the retention time and peak area of the extracted ions were within the range of 0.07%–1.14% and 1.21%–11.25%, respectively (Supplementary Table S3), proving QC samples were stable during the analytical procedure.

#### 3.4.3 Multivariate statistical analysis

**FIGURE 7:**
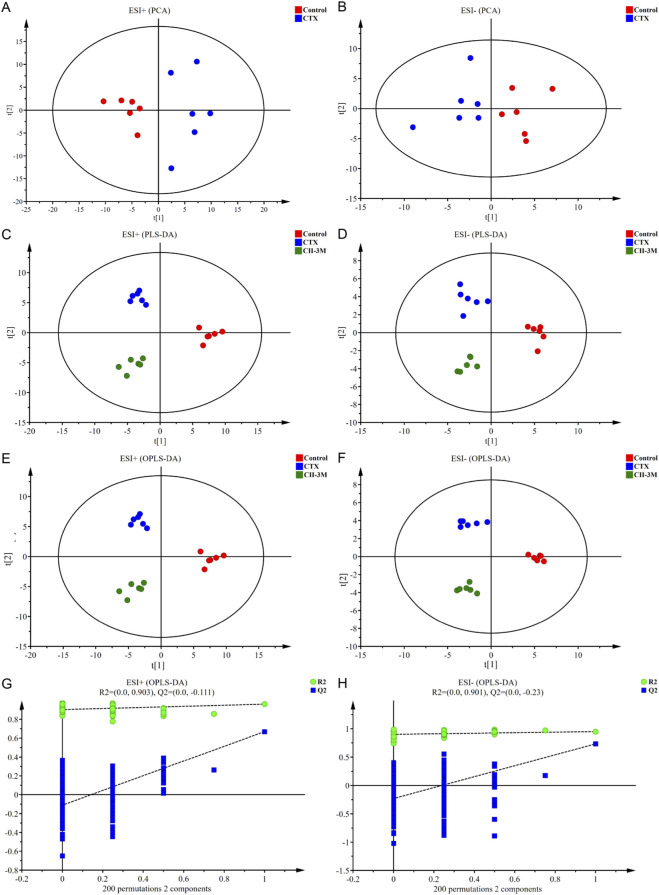
The results of multivariate statistical analysis. **(A,B)** PCA score plots based on the serum metabolic profiling of the control and model groups in positive and negative ion modes, ESI^+^: R^2^ = 0.501, ESI^−^: R^2^ = 0.541. **(C,D)** PLS-DA score plots of the control, model and CII-3M groups, ESI^+^: R^2^X = 0.501, R^2^Y = 0.962, Q^2^ = 0.545; ESI^−^: R^2^X = 0.539, R^2^Y = 0.932, Q^2^ = 0.635. **(E,F)** OPLS-DA score plots of the control, model and CII-3M groups, ESI^+^: R^2^X = 0.501, R^2^Y = 0.962, Q^2^ = 0.509; ESI^−^: R^2^X = 0.605, R^2^Y = 0.970, Q^2^ = 0.620. **(G,H)** Permutation test of the OPLS-DA models, ESI^+^: the intercepts of R^2^ = 0.903 and Q^2^ = −0.111, ESI^−^: R^2^ = 0.901, Q^2^ = −0.230.

A PCA score plot model between ESI^+^ and ESI^−^ was constructed, and the control rats and the CTX-induced model rats were clearly distinguished (Figures 7A,B). The result illustrated that the endogenous metabolite profiles of model rats were substantially different from those of the healthy rats, implying that significant metabolic disturbances had occurred in the serum of model rats.

Besides, the rats of normal group, model group, and CII-3M group exhibited obvious classification trends in PLS-DA (Figures 7C,D), which demonstrated that there were significant differences in the metabolic environment of rats under different physiological conditions, and CII-3 treatments recovered the immunosuppressive status.

OPLS-DA model was constructed in Figures 7E,F, and the model exhibited desirable interpretation ability (R^2^X ≥ 0.5, R^2^Y ≥ 0.9) and prediction ability (Q^2^ ≥ 0.5). Additionally, the permutation test was conducted to test the over-fitting of OPLS-DA model. Permutation tests yielded the intercepts of R^2^ = 0.903 and Q^2^ = −0.111 in the positive ion mode and R^2^ = 0.901, Q^2^ = −0.230 in the negative ion mode (Figures 7G,H), the intercepts of Q^2^ regression lines were less than 0 and all permuted R^2^ values to the left of the intercept were lower than the original points to the right, manifesting that the OPLS-DA models were robust without over-fitting. OPLS-DA plots for normal, model, and CII-3M groups in positive and negative ion models were clearly distinguishable. It was inferred that the endogenous substance metabolisms of rat were obviously disrupted, and could employ the metabolic profiles to filter for differential metabolites.

#### 3.4.4 Differential metabolite analysis

Based on VIP >1 and *P* < 0.05, a total of 27 metabolites were screened as potential biomarkers (Table 1; Supplementary Figure S24), which may be associated with how CII-3 influenced the development of immunosuppression in rats. Among them, 16 metabolites in the model group were obviously upregulated compared with those in the control group. 14 metabolites in the CII-3M group were obviously upregulated compared with those in the model rats. The results of the metabolic profiling analysis were consistent with the pharmacodynamic experimental results based on the serum biochemical indexes. It was speculated that the mechanism of CII-3 agianst immunosuppression was related to changes of levels of the differential metabolites.

**TABLE 1:** The information of differential metabolites identified in CTX-induced immunosuppressive rat serum which were recovered by CII-3.

No.	Metabolites	*m/z*	t_R_/s	Molecular formula	KEGG ID	CTX vs. control			CII-3M vs. CTX			Mode
						VIP	FC	Trend	VIP	FC	Trend	
1	Glucose 6-phosphate	259.0218	82.9	C_6_H_13_O_9_P	C00092	2.02	0.41	↓^##^	1.37	1.47	↑^*^	ESI^-^
2	Nicotinamide	121.1159	90.8	C_6_H_6_N_2_O	C00153	1.70	0.47	↓^#^	1.37	1.18	↑	ESI^-^
3	Citrulline	174.0867	96.1	C_6_H_13_N_3_O_3_	C00327	1.69	0.61	↓^#^	1.76	1.01	↑	ESI^-^
4	Acetylcholine	146.1170	100.6	C_7_H_16_NO_2_	C01996	1.84	0.60	↓^#^	1.24	1.21	↑	ESI^+^
5	Cytosine	112.0504	103.4	C_4_H_5_N_3_O	C00380	1.93	2.56	↑^#^	2.01	0.99	↓	ESI^+^
6	N-Alpha-acetyllysine	189.1233	103.4	C_8_H_16_N_2_O_3_	C12989	1.72	0.87	↓^##^	2.23	1.84	↑^**^	ESI^+^
7	N6-Acetyl-L-lysine	189.1241	146.2	C_8_H_16_N_2_O_3_	C02727	2.20	1.17	↑^##^	2.16	3.03	↑^**^	ESI^+^
8	Nornicotine	148.0968	178.6	C_9_H_12_N_2_	C06524	2.20	0.82	↓^##^	1.66	1.07	↑	ESI^+^
9	Deoxyuridine	227.0662	242.6	C_9_H_12_N_2_O_5_	C00526	1.49	2.01	↑^#^	1.75	1.02	↑	ESI^-^
10	(R)-3-Hydroxybutyric acid	103.0383	245.0	C_4_H_8_O_3_	C01089	2.42	0.71	↓^##^	2.06	0.17	↓^*^	ESI^-^
11	L-kynurenine	209.0922	280.1	C_10_H_12_N_2_O_3_	C00328	2.08	4.02	↑^##^	1.31	0.71	↓	ESI^+^
12	6-Amino-6-deoxyfutalosine	413.1400	320.2	C_19_H_19_N_5_O_6_	C20773	1.60	1.65	↑^#^	1.31	0.83	↓	ESI^+^
13	4-Hydroxybenzaldehyde	123.0406	333.8	C_7_H_6_O_2_	C00633	2.28	2.39	↑^##^	1.26	0.72	↓	ESI^+^
14	Kynurenic acid	190.0508	346.0	C_10_H_7_NO_3_	C01717	1.71	0.47	↓^#^	1.34	1.21	↑	ESI^+^
15	4-Hydroxybenzoic acid	139.0180	431.9	C_7_H_6_O_3_	C00156	1.30	2.66	↑^#^	1.72	1.23	↑	ESI^+^
16	Adipate semialdehyde	130.0653	443.4	C_6_H_10_O_3_	C06102	1.39	1.05	↑^#^	1.88	0.79	↓^*^	ESI^+^
17	Taurohyocholate	516.2951	528.4	C_26_H_45_NO_7_S	C15516	2.30	5.38	↑^##^	1.95	0.67	↓	ESI^+^
18	Myristic acid	229.1798	678.3	C_14_H_28_O_2_	C06424	1.89	1.42	↑^##^	1.56	0.92	↓	ESI^+^
19	Prostaglandin F2a	353.4721	759.4	C_20_H_34_O_5_	C02314	1.88	13.83	↑^##^	2.32	1.36	↑	ESI^-^
20	Bilirubin	585.2671	802.3	C_33_H_36_N_4_O_6_	C00486	2.04	1.77	↑^##^	2.53	1.48	↑	ESI^+^
21	5a-Cholesta-7,24-dien-3b-ol	384.3462	804.9	C_27_H_44_O	C05439	1.98	1.29	↑^##^	1.62	0.97	↓	ESI^+^
22	Lanosterin	425.2576	823.2	C_30_H_50_O	C01724	1.81	0.37	↓^##^	1.76	0.96	↓	ESI^-^
23	trans-1,2-Cyclohexanediol	114.9322	870.7	C_6_H_12_O_2_	C03739	2.06	0.50	↓^##^	1.72	1.21	↑	ESI^-^
24	Protoporphyrin IX	563.2622	907.7	C_34_H_34_N_4_O_4_	C02191	1.79	2.78	↑^#^	1.53	0.78	↓	ESI^+^
25	Acetylcholine chloride	180.9719	926.9	C_7_H_16_NO_2_·Cl	C08201	1.52	2.13	↑^#^	1.89	0.97	↓	ESI^-^
26	Fluorouracil	130.0081	976.3	C_4_H_3_FN_2_O_2_	C07649	1.21	1.12	↑^#^	1.44	0.49	↓	ESI^+^
27	Tartaric acid	151.0365	982.7	C_4_H_6_O_6_	C00898	1.92	0.55	↓^#^	1.61	1.12	↑	ESI^+^

^#^ *P* < 0.05.

^##^ *P* < 0.01 compared with the control group.

^*^ *P* < 0.05.

^**^ *P* < 0.01 compared with the model group.

**FIGURE 8:**
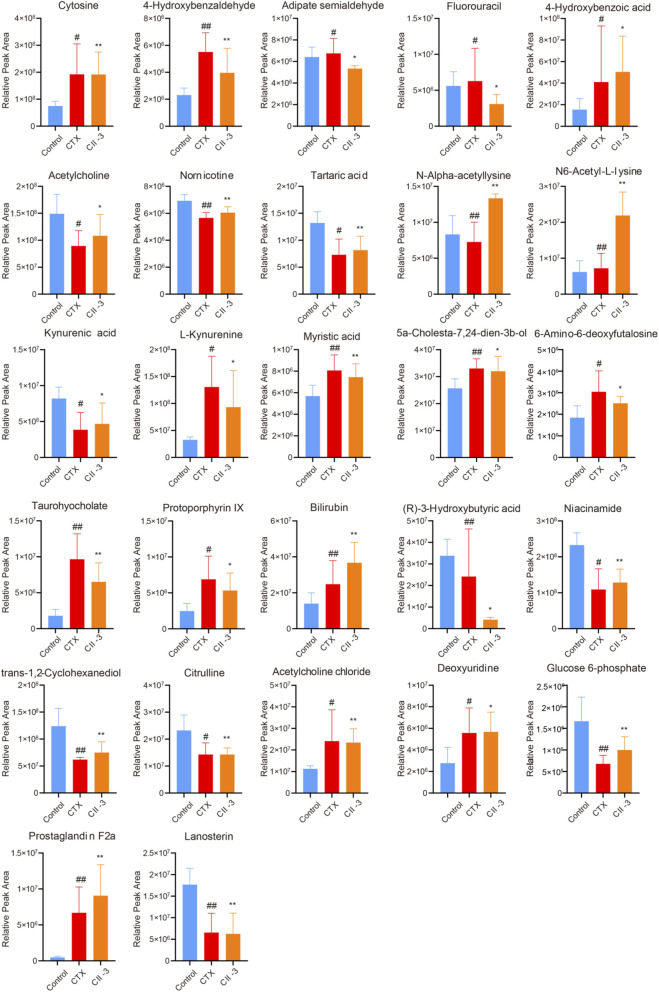
Comparison of relative abundance of differential metabolites with different groups of rats. Data are expressed as mean ± SD. ^#^ *P* < 0.05, ^##^ *P* < 0.01 compared with the control group; ^*^ *P* < 0.05, ^**^ *P* < 0.01 compared with the model group.

The dynamic trends of relative intensities of differential metabolites among different groups were presented in Figure 8, and the heatmap of representative differential metabolites were listed in Supplementary Figure S25. The results manifested that the model group was significantly different from the control group, and CII-3 treatment showed a tendency of correcting and reversing the variations of biomarkers in the abnormal metabolism of immunosuppression induced by CTX.

**FIGURE 9:**
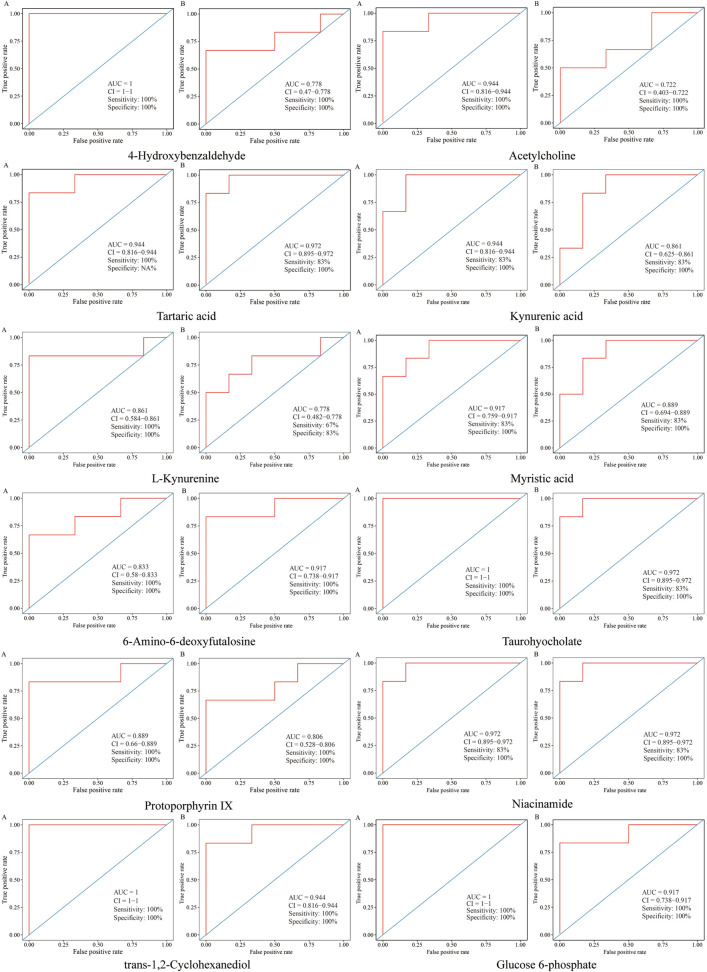
ROC curves for evaluation the predictive ability of the biomarkers in different groups. **(A)** ROC curves of metabolites between model and control groups. **(B)** ROC curves of metabolites between CII-3M and model groups.

Receiver operating characteristic (ROC) curve was constructed to evaluate the predictive capability of the metabolite markers, as disclosed in Figure 9), the area under curves (AUCs) of metabolites were greater than 0.70, most of them, such as kynurenic acid, tartaric acid, myristic acid, 6-amino-6-deoxyfutalosine, taurohyocholate, protoporphyrin IX, nicotinamide, trans-1,2-cyclohexanediol and glucose 6-phosphate were above 0.85, illustrating that these biomarkers were highly exclusive and sensitive.

#### 3.4.5 Metabolic pathway analysis

**FIGURE 10:**
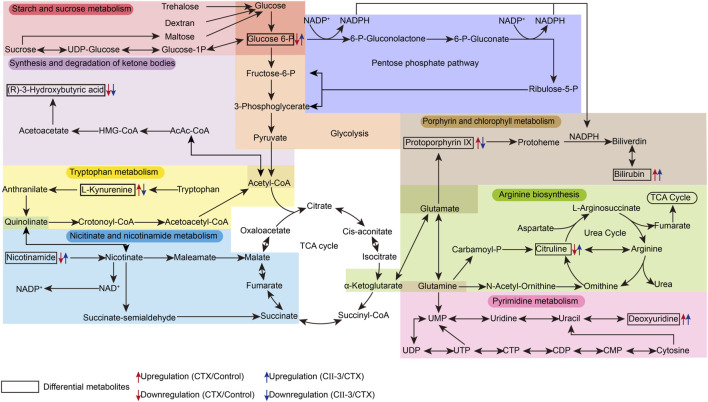
An overview of the interaction of regulatory pathways in the metabolic network for CII-3.

To investigate the potential pathways of CII-3 effect on CTX, 27 differential metabolites were entered into MetaboAnalyst 5.0 for pathway analysis. The results elucidated that CII-3-mediated amelioration of immunosuppression in rats was linked primarily to reversing the abnormality of starch and sucrose metabolism, arginine biosynthesis, steroid biosynthesis, porphyrin and chlorophyll metabolism, nicotinate and nicotinamide metabolism and tryptophan metabolism (impact value >0.1, Supplementary Figure S26). A metabolic pathway network diagram associated with biomarkers was established in Figure 10, which may be the core metabolic pathways of pharmacological effects of CII-3.

### 3.5 Correlation analysis of differential metabolites and efficacy indicators

Spearman correlation matrix analysis between efficacy indicators from pharmacodynamics experiments and potential biomarkers from metabolomics analysis were utilized to understand the intervention effect of CII-3 on immunosuppressed rats. Spearman analysis (Supplementary Figure S27) demonstrated close correlations between the potential biomarkers and biochemical indicators. Cytosine, 4-hydroxybenzaldehyde, L-kynurenine, myristic acid, 5a-cholesta-7,24-dien-3b-ol, 6-amino-6-deoxyfutalosine, taurohyocholate, protoporphyrin IX, bilirubin, acetylcholine chloride, deoxyuridine and prostaglandin F2a exhibited negative correlations with efficacy parameters, while acetylcholine, nornicotine, tartaric acid, kynurenic acid, niacinamide, trans-1,2-cyclohexanediol, citrulline, glucose 6-phosphate and lanosterin were positively correlated with efficacy parameters. Additionally, among the 27 metabolic biomarkers, the positive correlation between trans-1,2-cyclohexanediol and glucose 6-phosphate was strong, and the negative correlation of niacinamide, (R)-3-hydroxybutyric acid with prostaglandin F2a was strong, suggesting that differential metabolites were interrelated. These findings indicated that the changes of these differential metabolites mentioned above were closely participated in the protective effects of CII-3.

### 3.6 Integrated analysis of metabolomics and network pharmacology

**FIGURE 11:**
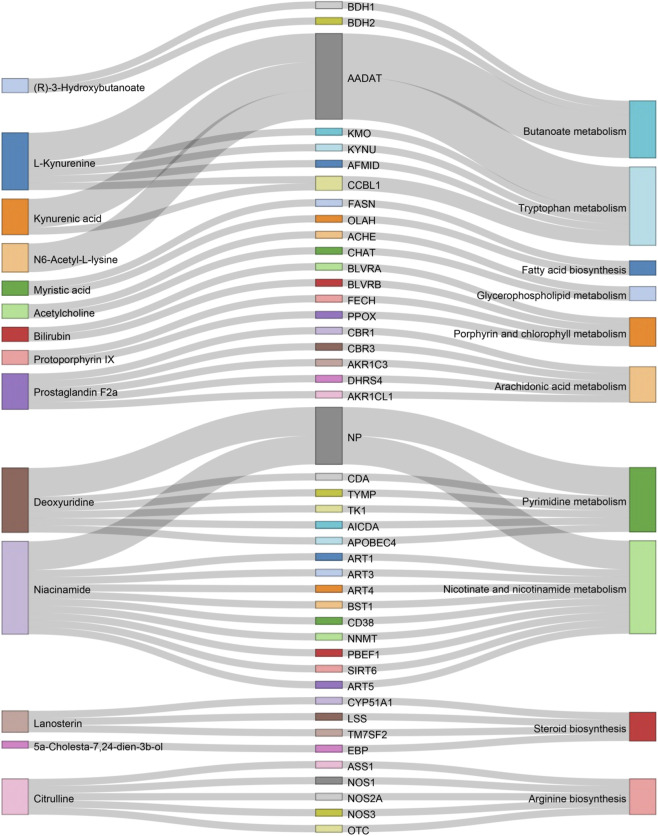
Sankey diagram of differential metabolites and associated genes and pathways. This sankey diagram, derived from Metscape results, illustrates the relationships between differential metabolites (left), genes (middle), and pathways (right).

**FIGURE 12:**
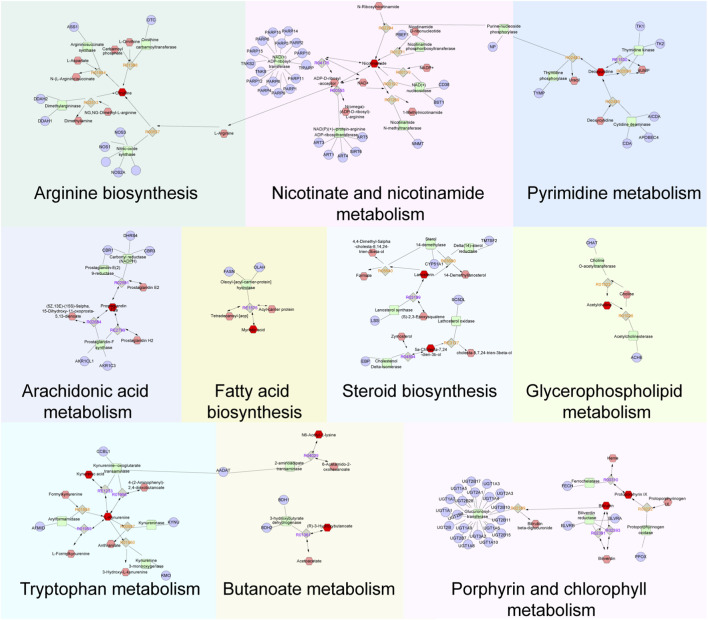
Metabolites-reactions-enzymes-genes-components network diagram. The network diagram illustrates the relationships among differential metabolites, reactions, enzymes, genes, and components. Dark red hexagons represent differential metabolites, light red hexagons represent compounds, gray diamonds represent reactions, green round rectangles represent proteins (enzymes), and purple circles represent genes. Different background colors indicate various pathways.

A total of 89 relevant metabolite targets were extracted using the MetScape plugin in the Cytoscape, and 110 intersectional targets were obtained from network pharmacology. In order to explore a comprehensive view of the effect of CII-3 on the improvement of immunosuppression, interaction network of “metabolites-reactions-enzymes-targets-components” was designed (Figures 11, 12).

**FIGURE 13:**
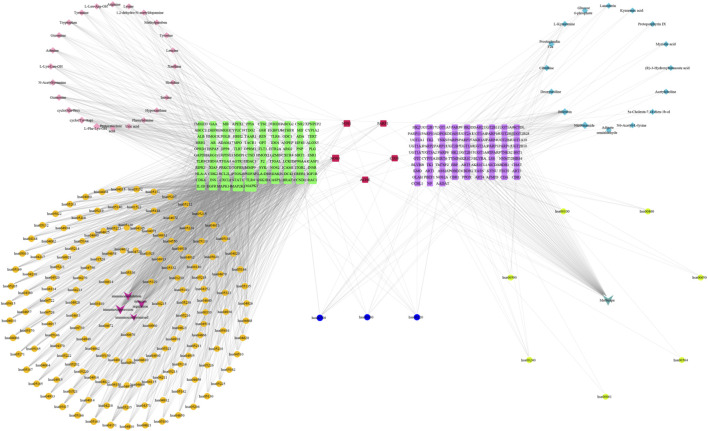
Components-targets-metabolites network based on metabolomics and network pharmacology. Red squares represent common targets between the intersection targets screened by the network pharmacology and differential metabolites targets; purple squares represent differential metabolites targets; green squares represent the intersection targets screened by the network pharmacology; primary blue ellipses represent common pathways; orange ellipses represent network pharmacology pathway codes; fluorescent yellow ellipses represent metabolomics pathway codes; pink diamonds represent CII-3 chemical ingredients; aqua blue diamonds represent differential metabolites; dark pink represent disease names; turquoise represent MetScape.

In addition,“components-targets-metabolites” network was constructed based on metabolomics and network pharmacology. The results (Figure 13) indicated that five genes, NOS1, NOS3, ACHE, CD38 and PARP1, were common targets both in network pharmacology and metabolomics, suggesting that CII-3 might act synergistically on these targets to play a therapeutic role. It was speculated that the ability of activaing immune response of CII-3 might be closely associated with these crucial targets, thereby modulating the levels of main metabolites such as citrulline through the relevant metabolic pathways, ameliorating immunosuppression consequently.

### 3.7 Molecular docking results

**FIGURE 14:**
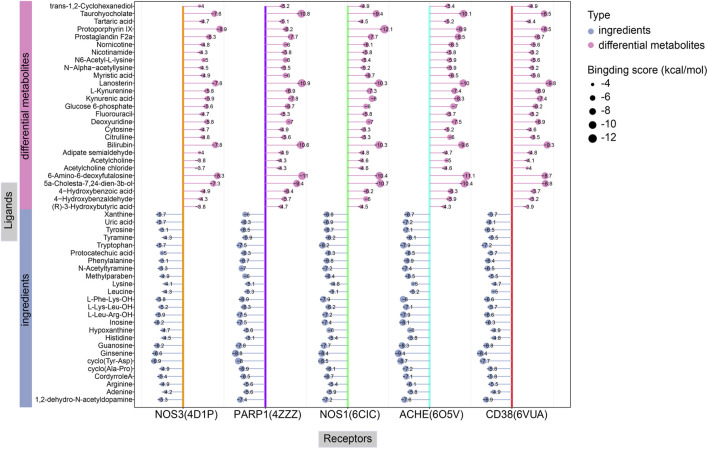
The binding energy for molecular docking between components of CII-3 *in vitro* and *in vivo* with five crucial targets.

**FIGURE 15:**
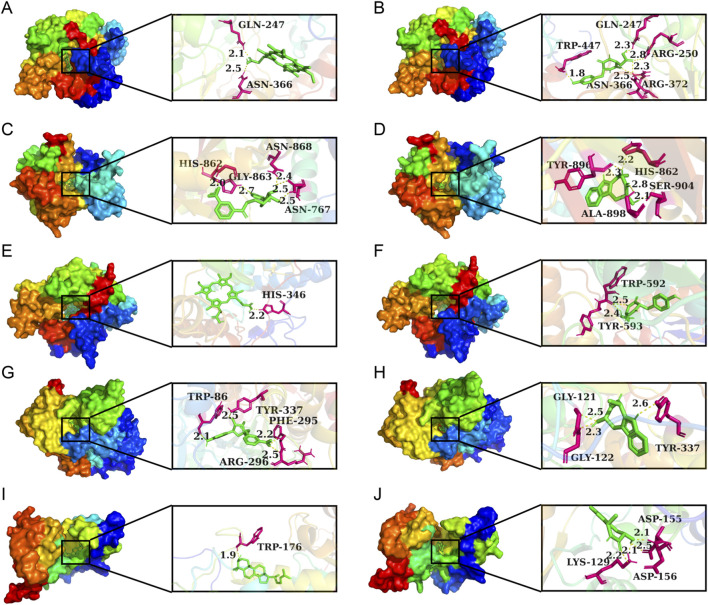
Molecular docking analysis of small molecules with core proteins. **(A)** NOS3-Protoporphyrin IX; **(B)** NOS3-cyclo (Tyr−Asp); **(C)** PARP1-6-Amino-6-deoxyfutalosine; **(D)** PARP1-Ginsenine; **(E)** NOS1-Protoporphyrin IX; **(F)** NOS1-cyclo (Tyr−Asp); **(G)** ACHE-Amino-6-deoxyfutalosine; **(H)** ACHE-Ginsenine; **(I)** CD38-Lanosterin; **(J)** CD38-Ginsenine. Green represents compounds, hot pink indicates potential docking sites, and yellow dashed lines signify hydrogen bonds.

Lower binding energies reflect stronger molecular interactions, with values below −7 kcal/mol generally indicating favorable binding activity. Figure 14 manifests the binding energies for components of CII-3 identified both *in vitro* (25 chemical ingredients characterized by UPLC-Q-TOF/MS) and *in vivo* (27 differential metabolites analyzed by UPLC-MS/MS) with five pivotal targets. The findings revealed that most of the components had low binding energies (≤-5 kcal/mol) with these targets. Accordingly, the top five ingredients *in vitro*, namely, ginsenine, cyclo (Tyr-Asp), guanosine, tryptophan and inosine with the lowest average binding energies of −8.32, −7.96, −7.36, −7.3 and −7.1 kcal/mol, respectively, were considered potential active components of CII-3 in counteracting immunosuppression. Figure 15 highlights representative molecular docking conformations corresponding to the lowest binding energy interaction between the five targets and components of CII-3 identified both *in vitro* and *in vivo*.

## 4 Discussion

Modern medicine has manifested that immunosuppression demonstrates as reductions of innate immunity and acquired immunity. In fact, an immunosuppressed individual has atrophic functional organs, changes in the numbers and functions of immune cells, and reduced levels and activities of cytokines. CTX-induced immunosuppression is a representative animal model used to evaluate immune enhancement *in vivo*. Serum IgG, IgM, IL-2, IL-6, WBC, RBC and PLT counts are important indicators, and the decreased levels of these parameters in the CTX group indicate existing immunity damage, which was confirmed by the pathological changes of spleen and thymus tissues. CII-3 extracted from *Periplaneta americana* L. is found to be a potent immune booster, which possesses favorable immunomodulatory properties by means of increasing immune organ index, restoring the normal histology and morphology of immune organs, promoting the number of WBC, RBC and PLT in rat blood, improving the secretion of immune-related cytokines and immunoglobulin levels in rat serum, and regulating IL-2 and IL-6 mRNA expression levels in rat spleen and thymus.

Three dose groups were incorporated in the animal experiment, yet the low-dose group failed to elicit significant regulatory effects on IL-6 and IgM. This observation could be attributed to the following reasons: First, the low dose may not have attained the threshold concentration necessary for exerting pharmacodynamic activity. Previous studies (Hossain et al., 2022) have reported that sub-threshold doses of immunomodulatory agents are incapable of inducing B cell receptor dimerization, which in turn abrogates IgM secretion. Second, rapid *in vivo* metabolism or clearance of the low-dose intervention might have led to insufficient duration of effective exposure. In contrast, medium and high doses maintained stable concentrations within the therapeutic window, thereby enabling sustained immune modulation. Third, endogenous negative immune feedback mechanisms may have counteracted the effects of the low dose, whereas higher doses were able to overcome this endogenous regulatory constraint and promote the production of IL-6 and IgM (Guryanova et al., 2022).

Bilirubin, a hydrophobic byproduct of haemoglobin breakdown found in bile, has been demonstrated to be a potent antioxidant, anti-inflammatory agent and immunomodulator (Bock, 2019). Unconjugated bilirubin was observed to interact with macrophages. What’s more, it plays a protective role during innate immunity related inflammation, the mechanism of which involves the control of the activation of NLRP3, AIM2, and NLRC4 inflammasomes and inhibition of the NF-κB signaling pathway (Li et al., 2020a). It was speculated that CII-3 might play a therapeutic role in immunosuppression by regulating inflammation, which needs to be confirmed by future research. However, certain trends that can provide reference for futher study were observed. The levels of IL-2 and IL-6 in serum of rats treated by CII-3 were significantly recovered compared with those in the model group, considering that CII-3 effectively exert immunomodulatory effect associated with the inhibition of inflammatory responses.

Citrulline functions as an important intermediate in the urea cycle and possesses anti-inflammatory and immunity elevation properties (Zezulová et al., 2016) by enhancing Nrf2 nuclear translocation and suppressing the NF-κB signaling pathway. In the current experiment, citrulline was found to be higher in the CII-3-treated rats than that in the CTX-treated rats, indicating that provision of CII-3 helps immune cells suppress arginine hydrolysis. Macrophages play a major role in the immune system, and macrophage biology is fundamentally driven by the phenotype of M1 and M2. M1 macrophages express the enzyme nitric oxide synthase (NOS1, NOS2 and NOS3), which metabolize arginine to nitric oxide and citrulline. It is generally believed that the increased citrulline is associated with a shift from glycolysis to oxidative phosphorylation (Ginguay and De Bandt, 2019). It may be speculated that CII-3 elevates the citrulline levels in the serum of immunocompromised rats by influencing the pathway of arginine biosynthesis, thereby promoting the secretion of IL-6 by M1 macrophages to improve immunity.

Lanosterin, the first sterol intermediate product in the cholesterol biosynthetic pathway, was identified as an endogenous immune regulators of macrophages in response to inflammatory stimuli (Zhang et al., 2021). Several studies (Araldi et al., 2017) have manifested that lanosterin is key substances in regulating cholesterol homeostasis and exerting immunological effects in macrophages. We tentatively speculated that CII-3 modulates the level of lanosterin by activating the steroid biosynthesis pathway, thereby exerting a regulatory effect on immunity, while, this speculation needs further study to confirm.

Tryptophan degradation by indoleamine 2,3-dioxygenase and tryptophan 2,3-dioxygenase yield L-kynurenine, and depletion of L-tryptophan and an increase in L-kynurenine exert important immunosuppressive effects by suppressing both T and NK cell functions. In addition, Schlichtner et al. reported L-kynurenine participates in cancer immune evasion by downregulating hypoxic signaling in T lymphocytes (Schlichtner et al., 2023). An increase in kynurenine level has also been reported in COVID-19 patients compared to healthy subjects (Kelly, 2020). In this study, we observed tryptophan metabolism with a marked reduction in serum level of L-kynurenine in CII-3 treatment group compared with the CTX group. The possible explanation for the result was that CII-3 inhibited the release of L-kynurenine to produce inhibitory effect on immunosuppression development. Besides, another metabolite identified in the current study, nicotinamide, is a promising tryptophan 2,3-dioxygenase inhibitor, which possesses anticancer property for the clinical therapy of some cancers (Badawy, 2018).

Nicotinamide, the active amide form of vitamin B3, which is cofactors in many redox reactions in cellular metabolism (Wei et al., 2019), is demonstrated to have the capacity to improve cellular energy metabolism, alleviate oxidative stress, maintain deoxyribonucleic acid (DNA) repair and gene stability, and regulate the immune microenvironment during inflammation. Nicotinamide has known immune-protective and cancer-preventive properties on its own or in combination with radical radiotherapy or cytotoxic drugs in humans (Yiasemides et al., 2009; Damian et al., 2010). Nicotinamide is decreased during immunosuppression, while an increase in nicotinamide level following CII-3 administration to ameliorate the immunocompromised state was detected in the current study.

Glucose-6-phosphate is abundant as a metabolic intermediate of glycolysis and as a substrate for glycogen synthesis (Tanaka et al., 2020). Glucose-6-phosphaten participates in the glycolytic pathway to generate pyruvate and produce adenosine triphosphate (ATP) for cellular activity as well as provide acetyl CoA for the tricarboxylic acid (TCA) cycle. Additionally, glucose-6-phosphaten also serves as a raw material for the pentose phosphate pathway to yield 5-phosphate ribose and nicotinamide adenine dinucleotide phosphate hydrogen (NADPH), NADPH can reduce oxidized glutathione, which plays a vital role in maintaining the redox state of cells. CTX administration causes an impairment of glycolysis via downregulation of glucose-6-phosphate. This impaired also weakens the ability for carbohydrate metabolism and lipid metabolism in CTX-induced rats. The intervention therapy of CII-3 in immunocompromised rats might promote the starch and sucrose metabolism and attenuate energy metabolism impairment by restoring the level of glucose-6-phosphate. In addition, the serum levels of IL-2 and IL-6 increased after CII-3 administration, it is confirmed that glucose-6-phosphate participates in the pentose phosphate pathway to produce NADPH, and therefore achieving immune regulation.

Jun et al. (2012) elucidated that neutrophils acquire intracellular glucose-6-phosphate or glucose through glucose-6-phosphate transporter or glucose-6-phosphatase-β to maintain glucose homeostasis. A deficiency in glucose-6-phosphate or glucose impairs neutrophil energy homeostasis and function characterized by reduced intracellular levels of glucose-6-phosphate, ATP, and NADPH, and eventually leads to neutrophil dysfunction. Our study showed that CII-3 can restore the WBC level in the peripheral blood of immunocompromised rats, the result illustrated that CII-3 treatment might regenerate the energy homeostasis of neutrophils mainly through the upregulation of glucose-6-phosphate. However, these findings needed to be further confirmed by next experiments.

The above research manifested that these metabolites were strongly associated with the efficacy indicators of immune. Furthermore, the effects of CII-3 in alleviating immunosuppression were attributed to the regulation of metabolite-related genes and the correction of metabolic disorders, mainly involving butanoate metabolism, tryptophan metabolism, fatty acid biosynthesis, glycerophospholipid metabolism, porphyrin and chlorophyll metabolism, pyrimidine metabolism, nicotinate and nicotinamide metabolism, steroid biosynthesis and arginine biosynthesis.

Arginine biosynthesis is intricately linked to the immune system, for example, activated T- cells require increased arginine uptake and metabolism to support their clonal expansion during an immune response. Additionally, arginine metabolism in macrophages can influence their cytokine production profile, thereby regulating the inflammatory response. What’s more, arginine -derived metabolites contribute to wound healing, which is an important part of the body’s immune-mediated response to injury. It promotes the synthesis of collagen and other extracellular matrix components, aids in angiogenesis, and attracts immune cells and fibroblasts to the wound site, facilitating the repair process (Feng et al., 2025).

Adequate porphyrin metabolism is necessary to ensure the proper function of immune- related proteins, which is crucial for oxygen transport, energy production, and redox reactions in immune cells. The breakdown products of porphyrin metabolism, such as biliverdin and bilirubin, have antioxidant properties and can protect macrophages from oxidative damage during phagocytosis, thereby enhancing their ability to clear pathogens.

Nicotinate and nicotinamide are precursors of nicotinamide adenine dinucleotide (NAD^+^) and its phosphorylated form (NADP^+^). These coenzymes are crucial for energy - generating metabolic pathways such as glycolysis, the tricarboxylic acid cycle, and oxidative phosphorylation in immune cells (Yang et al., 2024). Adequate NAD^+^ and NADP^+^ levels, maintained through nicotinate and nicotinamide metabolism, are essential to meet energy requirements, enabling the cells to carry out functions (Ashihara et al., 2011).

Tryptophan metabolism influences cytokine production. Some tryptophan metabolites can regulate the production of pro-inflammatory IL-6 and TNF-α. Tryptophan metabolites also affect macrophage polarization. They can drive macrophages to adopt an anti-inflammatory M2- like phenotype, which is involved in tissue repair and immune-response resolution (Chen et al., 2022).

Fatty acid biosynthesis provides the necessary fatty acids for the synthesis of cell membranes in immune cells, and the proper incorporation of specific fatty acids into the membrane of T-cells and B-cells is essential for the efficient recognition of antigens and the activation of immune signaling pathways (Dieckmann, 2024). What’s more, fatty acids can be broken down through beta-oxidation to generate ATP, which is required for processes such as cell proliferation, cytokine production, and phagocytosis. Adequate fatty acid biosynthesis is therefore necessary to meet the energy needs of immune cells.

Activated T-cells upregulate the expression of enzymes involved in pyrimidine biosynthesis to support their rapid proliferation. Deficiencies in pyrimidine metabolism can lead to impaired immune cell proliferation and a weakened immune response. Some studies (Lyu et al., 2024) have shown that certain pyrimidine-related compounds can modulate the expression and secretion of cytokines such as IL-2, IFN-γ and TNF-α, which are key mediators of immune responses.

The pivotal genes obtained in this study were also closely related to immunoregulatory responses. NOS1 is prevalent in neuronal tissue, and can also be expressed in macrophages. The NO produced by NOS1 inhibits Th1/Th17 differentiation while promoting regulatory T cell (Treg) expansion, thereby maintaining immune tolerance. Studies have shown that deletion of NOS1 leads to downregulation of innate immune (Grohmann et al., 2017; Wijnands and Castermans, 2015). Recent studies (Chen et al., 2022) also link NOS1 to metabolic reprogramming in immune cells, where NO-dependent inhibition of mitochondrial respiration drives glycolytic shifts in activated macrophages.

NOS3, primarily expressed in endothelial cells and immune cells, plays a vital role in vascular-immune crosstalk (Król and Kepinska, 2020). Endothelial NOS3-derived NO maintains vascular integrity by suppressing adhesion molecules (e.g., ICAM-1) and leukocyte recruitment, thereby limiting acute inflammation. NOS3 is also involved in the regulation of adaptive immunity: NO produced under the action of NOS3 promotes the homing of naive T cells to lymph nodes via upregulating CCR7, while it also dampens the effector functions of cytotoxic T cells (CTLs) by means of inhibiting granzyme B secretion (Lisi et al., 2021; Gantner BN, LaFond and Bonini, 2020).

CD38, a multifunctional glycoprotein, modulates immunity through NAD+ metabolism and receptor signaling. CD38 is central regulator of T cell, mediating the cytokine secretion and immune synapse formation, thereby assisting T cells in executing immune responses (Li et al., 2020b; Glaría and Valledor, 2020; Kar et al., 2020). CD38 exerts an influence on the functions of B cells and NK cells as well. It facilitates the process of class-switch recombination in B cells, and boosts the cytotoxicity of NK cells by upregulating perforin expression (Zhu et al., 2020; Dwivedi and Rendón-Huerta, 2021; Ghorani et al., 2023).

ACHE, traditionally known for hydrolyzing acetylcholine, regulates immunity through the cholinergic anti-inflammatory pathway and direct immune cell interactions. Recent investigations underscores the role of ACHE in macrophage metabolic reprogramming: its overexpression promotes M1 macrophage glycolysis, while its inhibition directs M2 macrophages toward oxidative phosphorylation (Singh et al., 2025; Kattner et al., 2023).

PARP1, a DNA damage sensor, regulates immunity through chromatin remodeling and cell death pathways. In innate immunity, PARP1 increases TLR-induced cytokine production by PARylating histones at NF-κB target genes but limits cGAS-STING signaling to prevent excessive type I interferon responses. PARP1 deficiency in T cells impairs DNA repair, leading to apoptosis and defective adaptive immunity (Laudisi et al., 2011; Wang et al., 2022).

To investigate the material basis of CII-3 against immunosuppression, the main compositions of CII-3, which included amino acids, nucleosides, dipeptides and cyclic peptides, purines were identified. To explore the potential mechanisms of immunoregulatory property of CII-3, metabolomics, network pharmacology and molecular docking were performed. These findings manifested that the immunomodulatory activity of CII-3 might be attributed to affect metabolite-related genes (NOS1, NOS3, ACHE, CD38 and PARP1) through multiple metabolic pathways (butanoate metabolism, fatty acid biosynthesis, glycerophospholipid metabolism, arachidonic acid metabolism, porphyrin and chlorophyll metabolism, pyrimidine metabolism, steroid biosynthesis, tryptophan metabolism, arginine biosynthesis and nicotinate and nicotinamide metabolism), leading to the change of efficacy-related biomarkers (citrulline, nicotinamide, deoxyuridine, acetylcholine, lanosterin, 5a-Cholesta-7,24-dien-3b-ol, myristic acid, prostaglandin F2a, kynurenic acid, L-kynurenine, (R)-3-Hydroxybutyric acid, protoporphyrin IX, glucose-6-phosphate and bilirubin). Which may be the core mechanism of CII-3 in the treatment of immunosuppression induced by CTX.

There are some limitations in the current study. First, the detailed relationship among bioactive components, targets, signal pathways and metabolic pathways have not been comprehensively uncovered. Second, although this study provides a preliminary indication of the potential mechanisms of CII-3 against immunosuppression, all speculations herein require further confirmation by experimental verification (e.g., *in vitro* or *in vivo* experiments including qRT-PCR and western blotting), For instance, NOS1/NOS3 (associated with the arginine biosynthesis pathway) and CD38 (linked to the nicotinate/nicotinamide metabolism pathway) should be given priority consideration in subsequent verification. Third, owing to the limitation of the number of MS/MS spectra of medicinal insects in the public databases, there remain several compositions of CII-3 cannot be qualitatively characterized, resulting in a few number of bioactive components and targets of CII-3 were not screened out by the network pharmacology, in fact, CII-3 regulates more than just the five hub targets mentioned, more metabolites and other substances may participate in the effect of CII-3 on the improvement of immunosuppression, frontier methods and techniques (i.e., multi-omics) should be employed in large studies in the follow-up reaearch. In addition, the specific components of CII-3 responsible for immunomodulatory effect remain unidentified, warranting subsequent investigation into its active monomeric compounds.

## 5 Conclusion

In this study, a sensitive and reliable UPLC-Q-TOF/MS method was established for structural elucidation of multiple chemical components of CII-3. It laid a solid foundation for the clarification of chemical material basis information of CII-3 and provides important data for the further in-depth study of the active constituents for pharmacological activities.

To our knowledge, this is the first study to delineate the immune-enhancing effects and related mechanism of CII-3 in immunosuppressed rat model by integrating metabolomics, network pharmacology and molecular docking. Our study uncovered that CII-3 exerts potential immunomodulatory activity for immunosuppression therapy by its multiple chemical constitutes modulating multiple targets, multiple metabolites and multiple pathways, and this research shed new light on the therapeutic benefit of immune modulation for the utilization and promotion of *Periplaneta americana L*. resources.

## Data Availability

The original contributions presented in the study are included in the article/Supplementary Material, further inquiries can be directed to the corresponding authors.
