# Supplementary material for: Deciphering the immunomodulatory mechanisms of Periplaneta americana L. extract CII-3: insight from integrated metabolomics and network pharmacology
Source: Front Cell Dev Biol. 2026 Jan 7;13:1718560. doi: 10.3389/fcell.2025.1718560 (PMC12819709; doi:10.3389/fcell.2025.1718560)
Supplement: Supplementary file 1 [file DataSheet1.docx]

***Supplementary Material***

**Deciphering the Immunomodulatory Mechanisms of *Periplaneta americana* L. Extract CⅡ-3: Insight from Integrated Metabolomics and Network Pharmacology**

Yilin Wang^1,2,3†^, Yingxiang Wu^1,2,3^^†^, Xi Liu^1,2,3^, Zhiyan Lu^1,2,3^, Tianqian Li^1,2,3^, Yang Jin^4^ , Yan Wang^1,2,3*^ and Jiali Zhu^1,2,3*^

^1^ Yunnan Provincial Key Laboratory of Entomological Biopharmaceutical R&D, Dali University, Dali, China

^2^ National-Local Joint Engineering Research Center of Entomoceutics, Dali University, Dali, China

^3^ School of Pharmacy, Dali University, Dali, China

^4^ Traditional Chinese Medicine Hospital of Dali,Dali, China

*** Correspondence:**Yan Wang,
🖂jessica9428@sina.com

Jiali Zhu,

🖂zjial_25@163.com

^†^These authors share first authorship

**Materials and reagents**

CTX was purchased from Baxter Oncology GmbH (catalog number: 1I493A, Hella, Germany). Levamisole (LM, catalog number: 201204, Guangdong, China) was purchased from Nanguo Pharmaceutical Co., Ltd. CⅡ-3 (Batch No. 20201112) was manufactured by SINOWAY Natural Pharmaceuticals Co., Ltd. (Kunming, China), and the preparation process was conducted according to the instructions provided by a national invention patent (CN200810059054.X). Briefly, air-dried *Periplaneta americana* L. powder, after being crushed, underwent extraction with ethanol. The resulting ethanol extract was concentrated and then cooled. Subsequently, the upper oil layer was removed and the remaining lower solution was subjected to macroporous resin column chromatography for separation, with elution carried out using alcohol/water mixtures to obtain the bioactive fraction CII-3. The reference substance lysine, arginine, tyrosine, tryptophan, phenylalanine, hypoxanthine, xanthine, adenine, inosine and guanosine were obtained from Sichuan Wei Keqi Biological Technology (catalog number: wkq22072006, wkq22072609, wkq22072201, wkq22040608, wkq22092706, wkq22060808, wkq22080307, wkq22080405, wkq22082501 and wkq22051905, respectively; purity≥98%). Rat enzyme-linked immunosorbent assay (ELISA) kits, including those for IL-2 (catalog number: H003-1-1; sensitivity: 2-600 ng/L), IL-6 (catalog number: H007-1-1; sensitivity: 2-600 ng/L), IgG (catalog number: H106-1-1; sensitivity: 0.5-100 mg/mL), and IgM (catalog number: H109-1-1; sensitivity: 20-6000 µg/mL), were provided by Nanjing Jiancheng Bioengineering Institute (Nanjing, China), 2-chloro-L-phenylalanine (catalog number: A190846) were purchased from Shanghai Aladdin Bio-Chem Technology Co., LTD (Shanghai, China). Acetonitrile (catalog number: A998-4) , methanol (catalog number: A452-4) and formic acid (catalog number: A117-50) of HPLC grade were purchased from Fisher Scientific (Fair Lawn, NJ, USA). Animal total RNA isolation kit (catalog number: DP451; sensitivity:6-10 μg/mg), fastking RT kit with gDNase (catalog number: KR116; sensitivity:50 ng-2 μg) and superreal premix plus SYBR Green (catalog number: FP205; sensitivity:10 pg-100 ng) were purchased from Tiangen Biotech Co., LTD (Beijing, China). All primers used in the current study were synthesized by Sangon Biotech Co., LTD (Shanghai, China). Other reagents were of analytical grade.

Table S1 The primer sequences used in this study.

| Rat gene | Primer sequences |
| --- | --- |
| β-actin | Forward primer(5’-3’) : **ACATCCGTAAAGACCTCTATGCC** |
|  | Reverse primer(5’-3’): **TACTCCTGCTTGCTGATCCAC** |
| IL-6 | Forward primer:(5’-3’) **AGAGACTTCCAGCCAGTTGC** |
|  | Reverse primer(5’-3’): **AGCCTCCGACTTGTGAAGTG** |
| IL-2 | Forward primer(5’-3’): GCAGGCCACAATTGAAAC |
|  | Reverse primer(5’-3’): CCAGCGTCTCTTCCAAGTGAA |


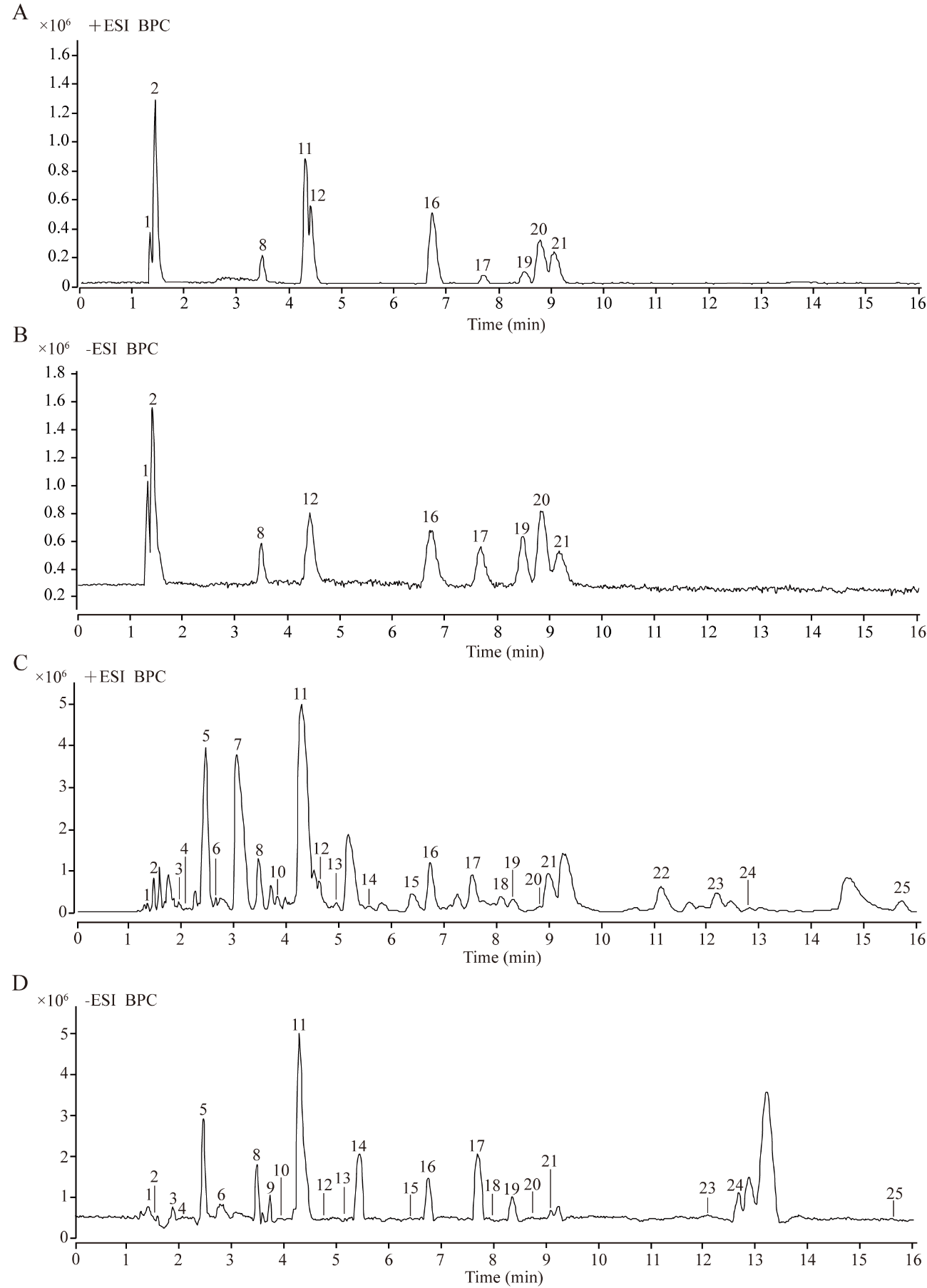


**Figure S1:** BPC of reference substances solution (A,B) and aqueous extract solution of CⅡ-3(C,D) by UPLC-Q-TOF/MS. (A,C) the positive ion mode; (B,D) the negative ion mode.


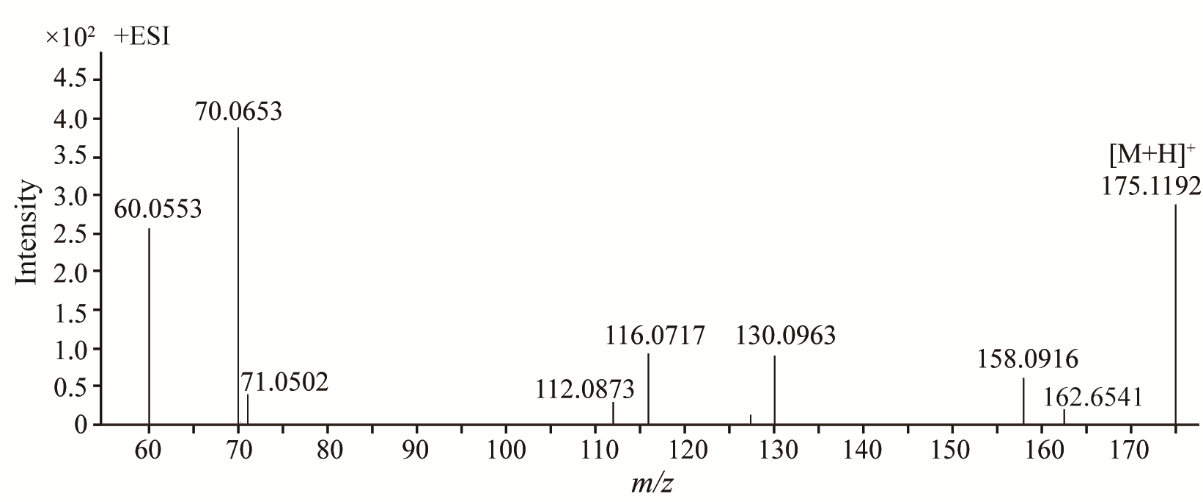


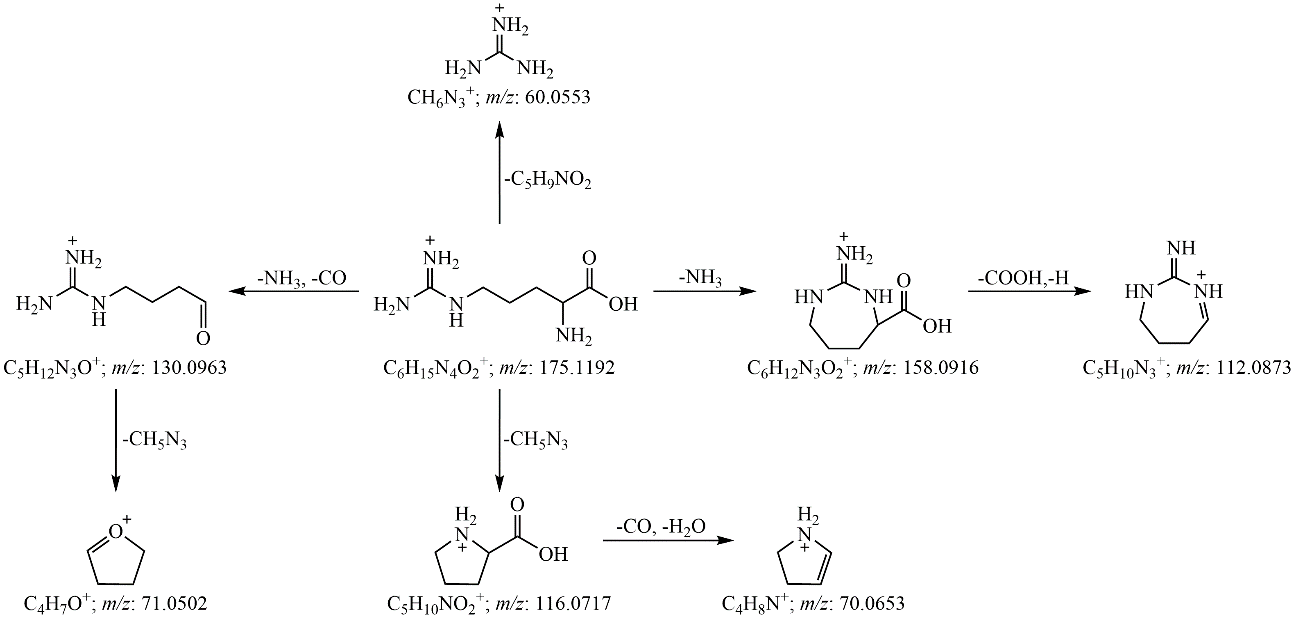


**Figure S2:**The MS/MS spectrum and proposed fragmentation pathways of arginine in the positive ion model (compound 2).


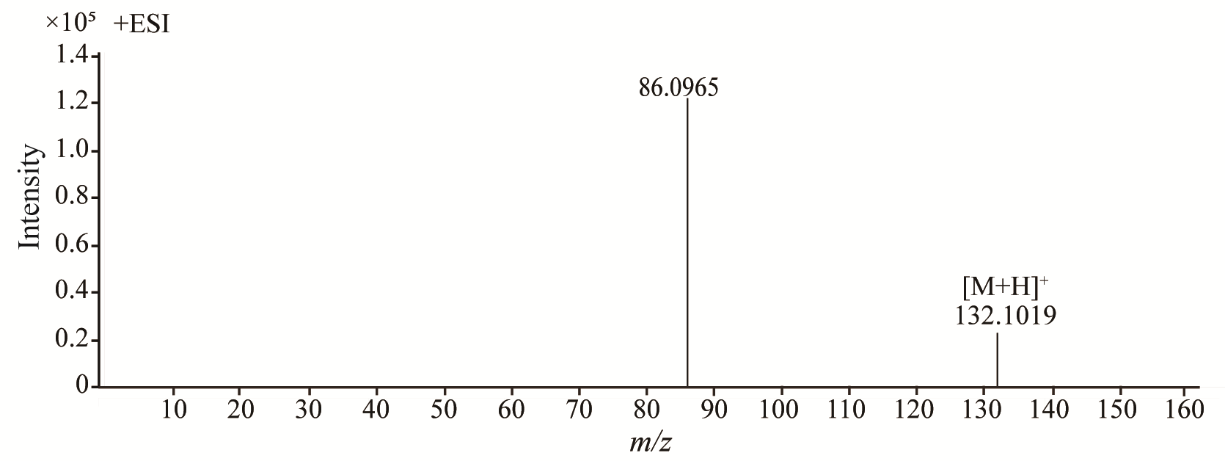


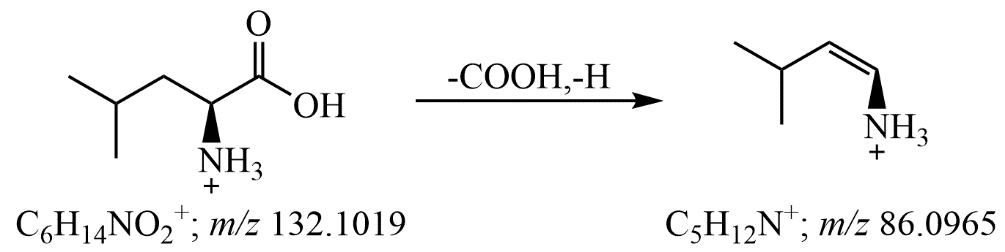


**Figure S3:**The MS/MS spectrum and proposed fragmentation pathway of leucine in the positive ion model (compound 5).


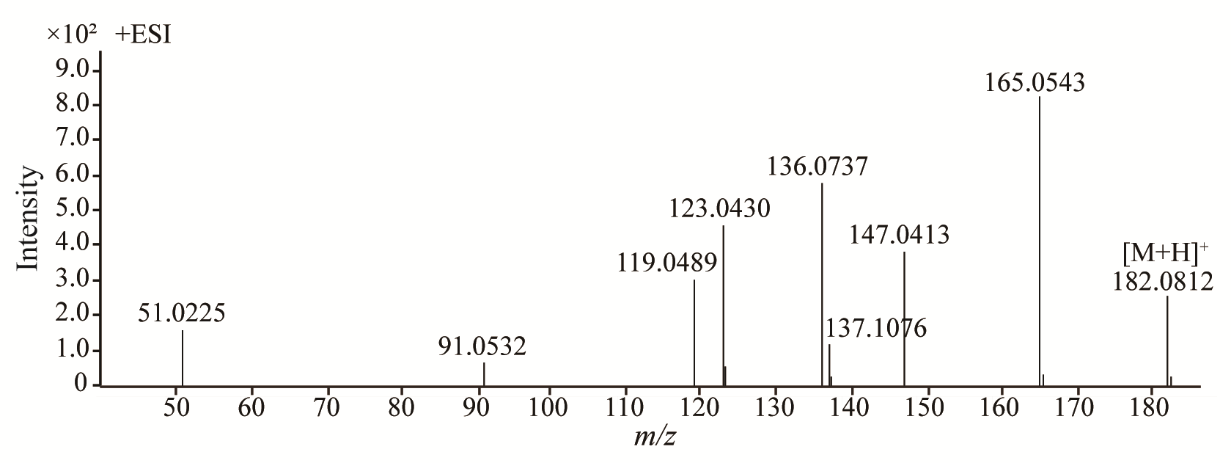


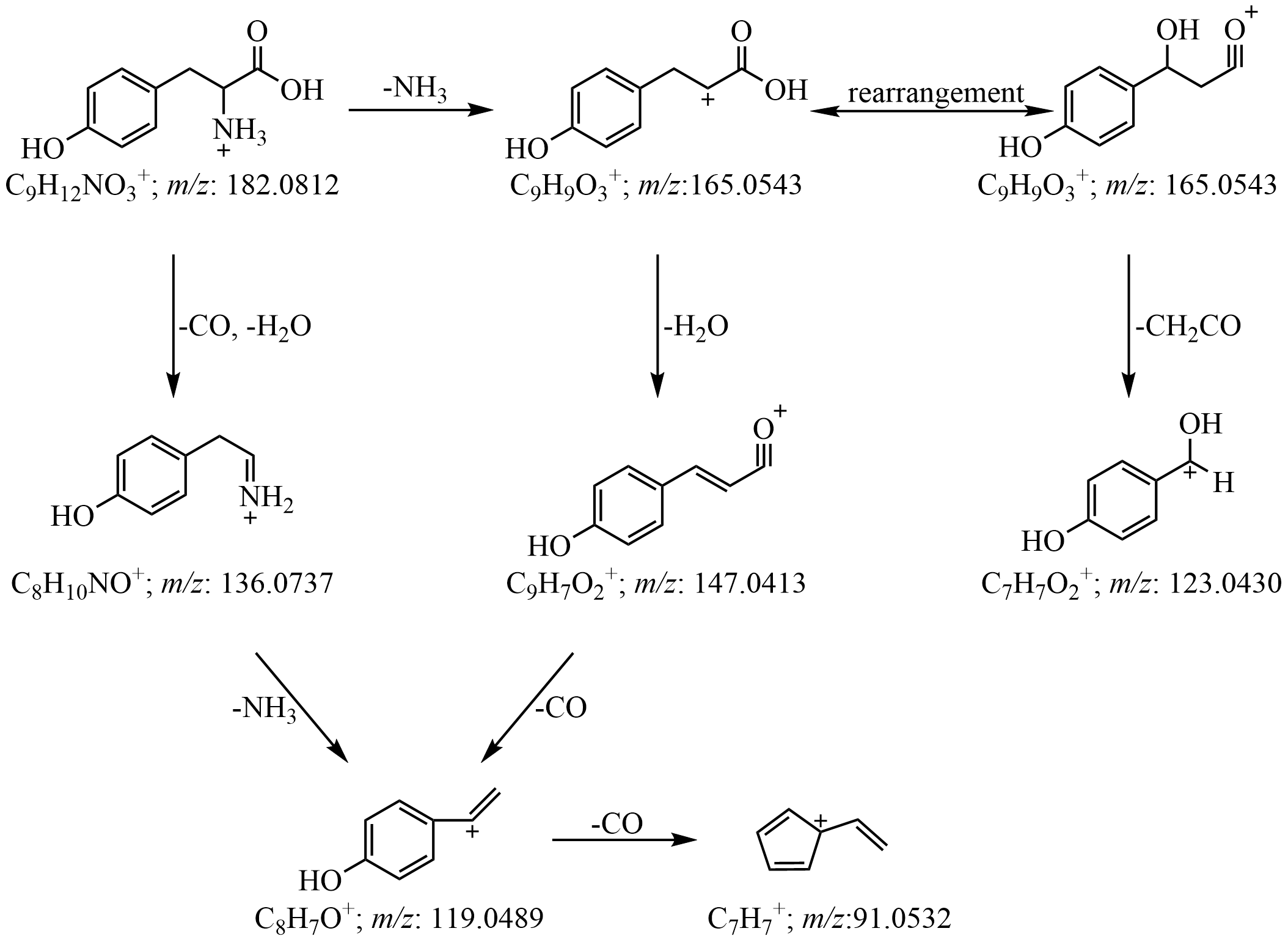


**Figure S4:**The MS/MS spectrum and proposed fragmentation pathways of tyrosine in the positive ion model (compound 8).


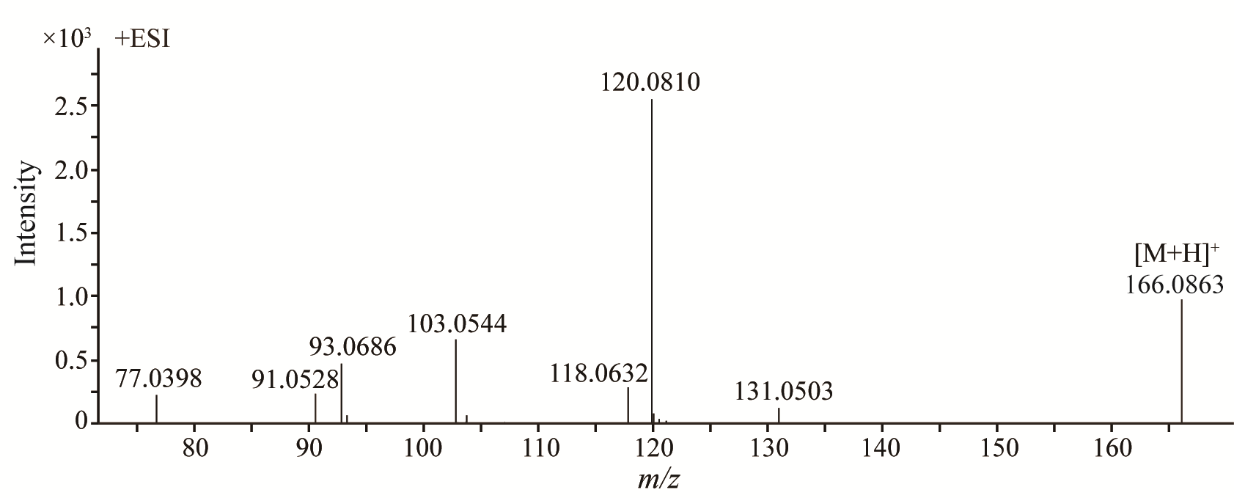


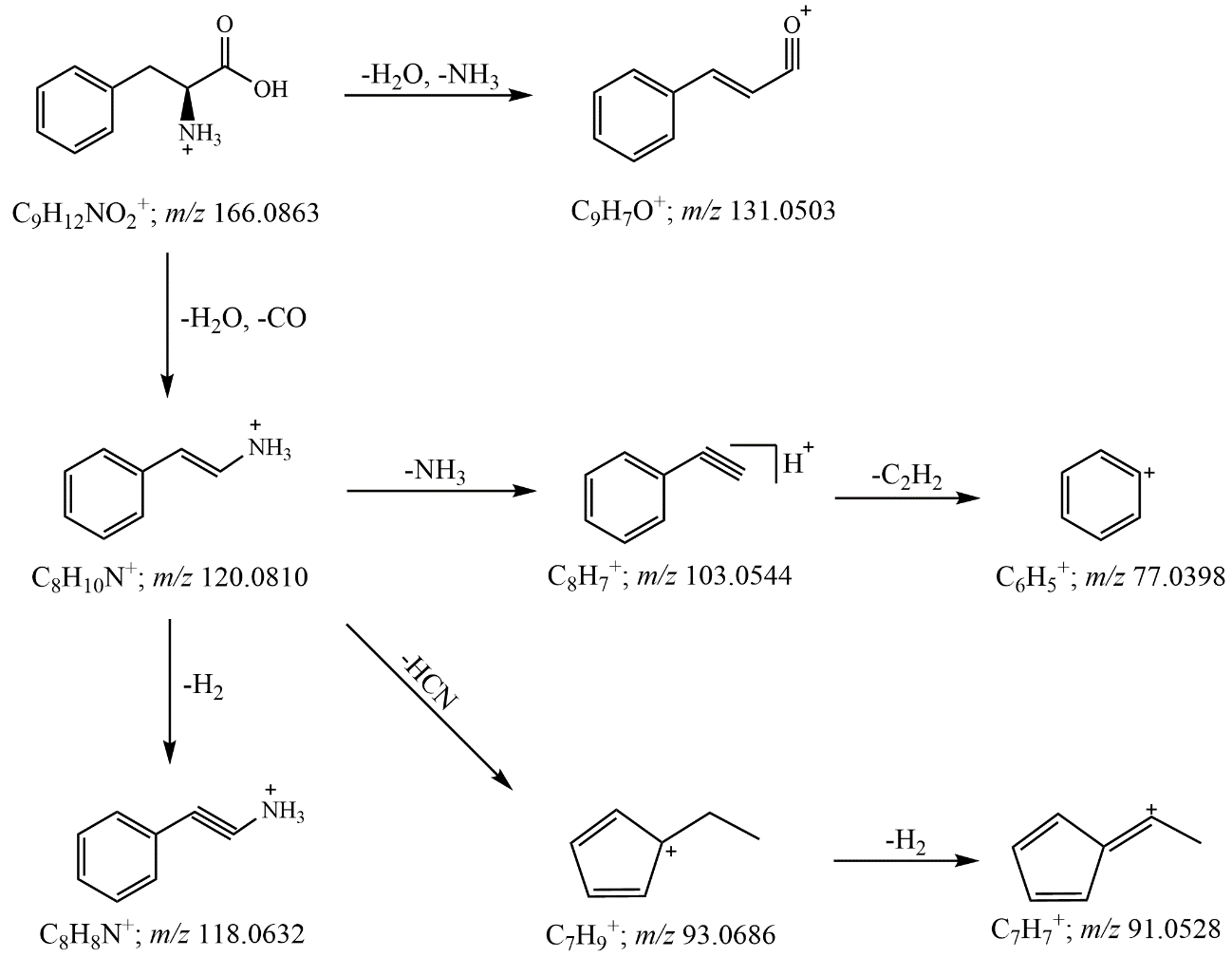


**Figure S5:**The MS/MS spectrum and proposed fragmentation pathways of phenylalanine in the positive ion model (compound 11).


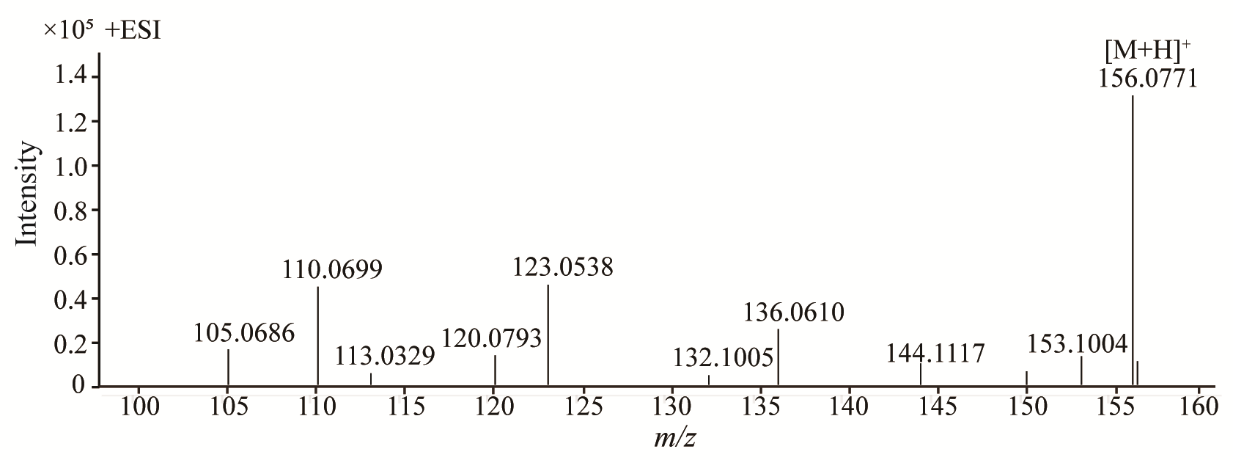


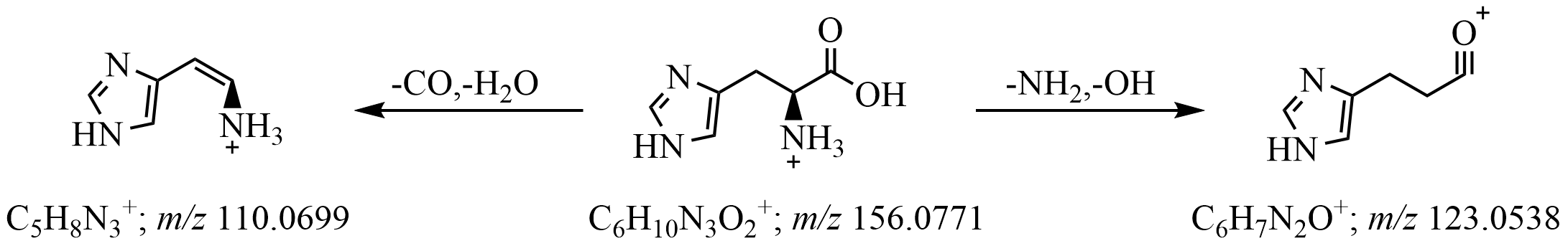


**Figure S6:**The MS/MS spectrum and proposed fragmentation pathways of histidine in the positive ion model (compound 13).


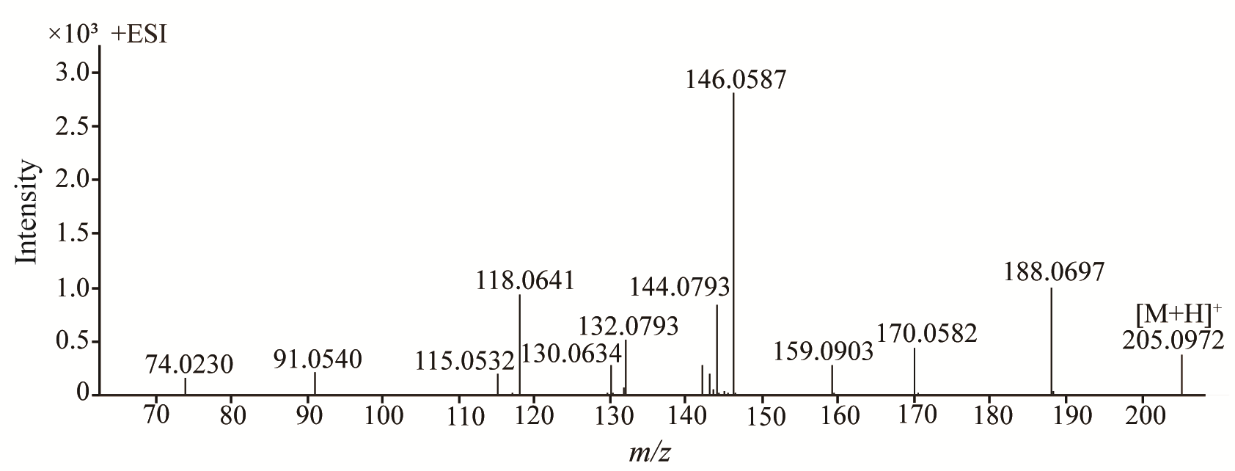


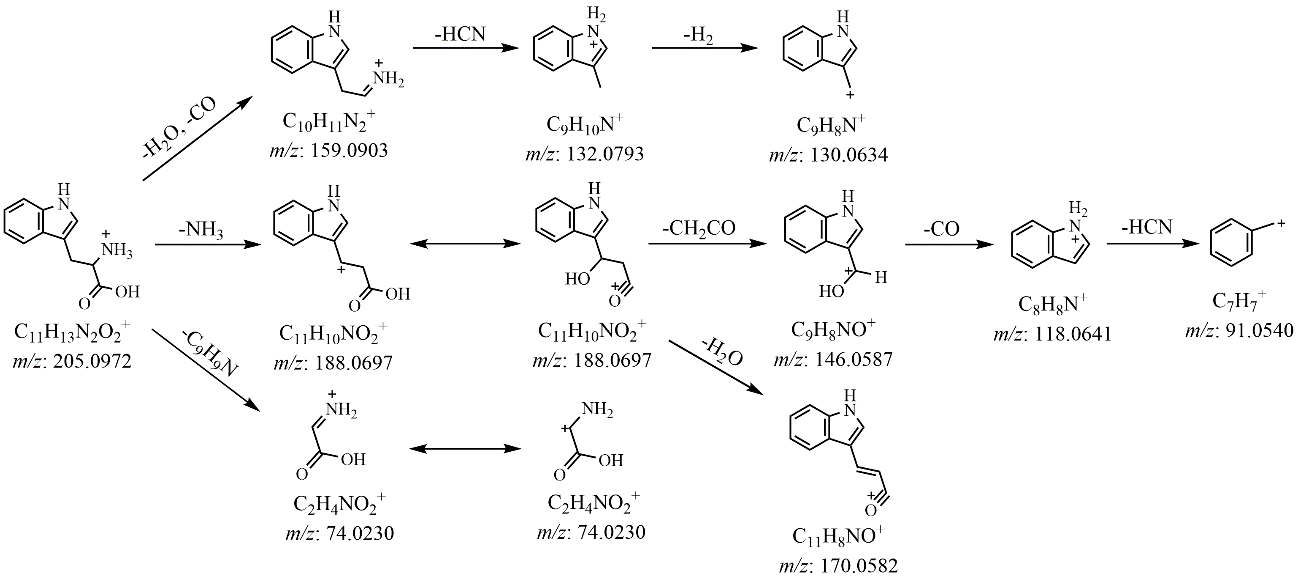


**Figure S7:**The MS/MS spectrum and proposed fragmentation pathways of tryptophan in the positive ion model (compound 21).


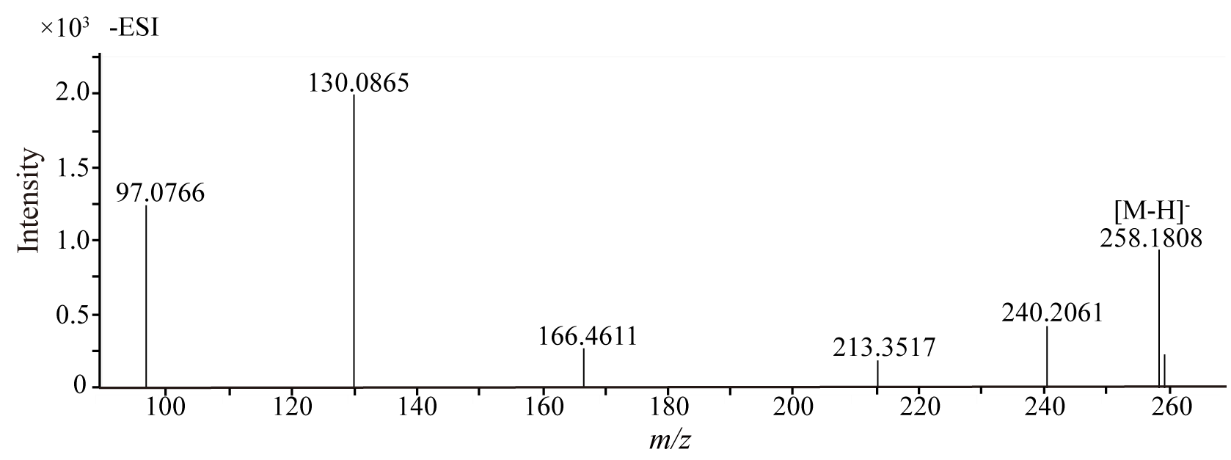


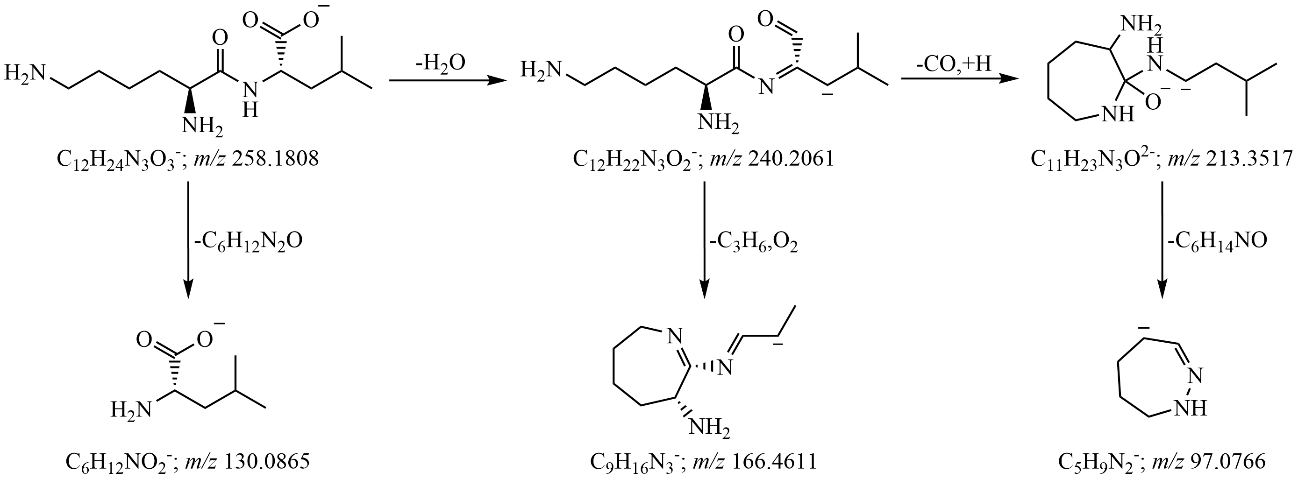


**Figure S8:**The MS/MS spectrum and proposed fragmentation pathways of L-Lys-Leu-OH in the negative ion model (compound 3).


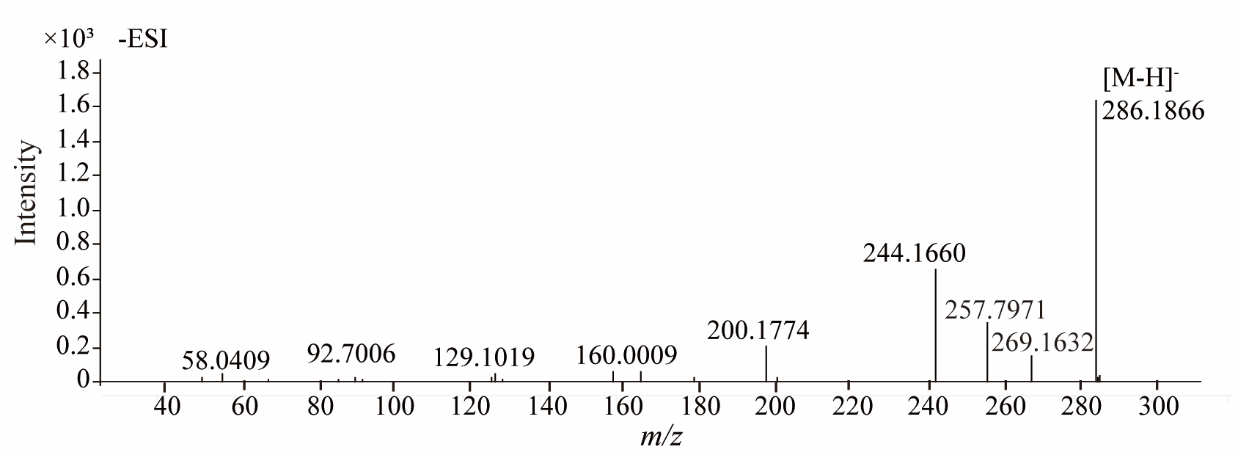


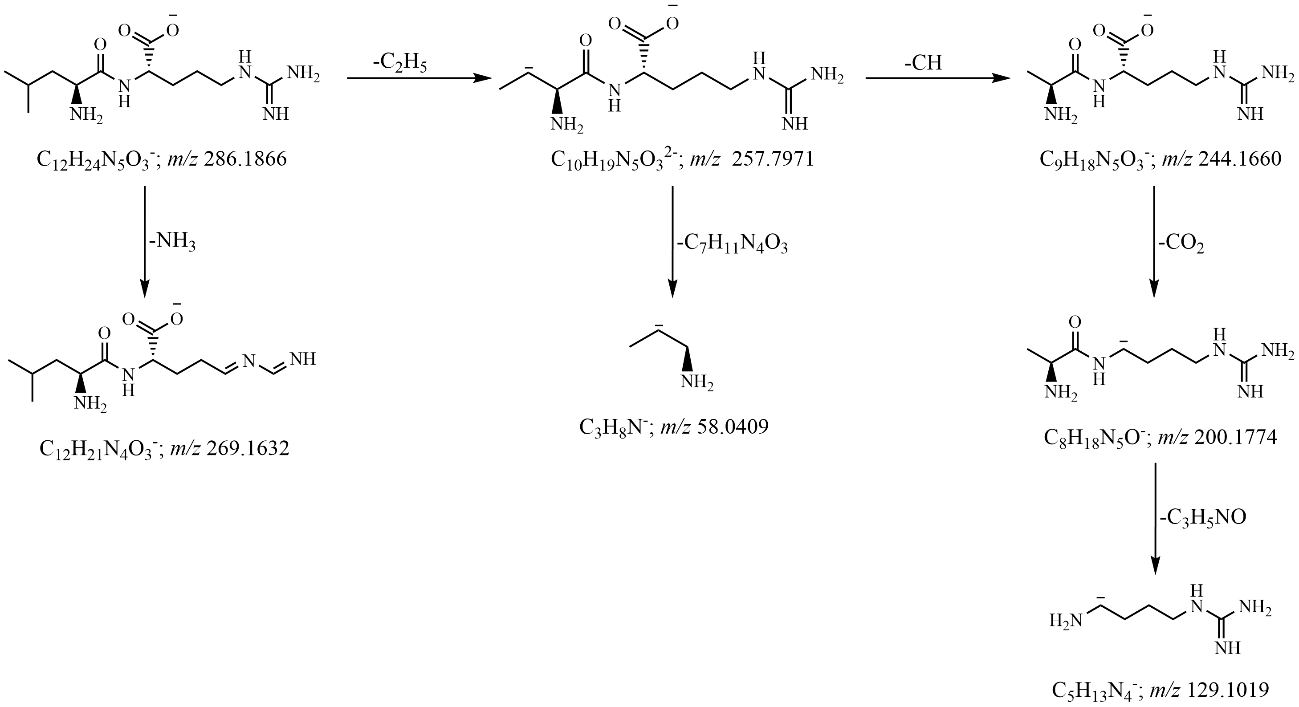


**Figure S9:**The MS/MS spectrum and proposed fragmentation pathways of L-Leu-Arg-OH in the negative ion model (compound 4).


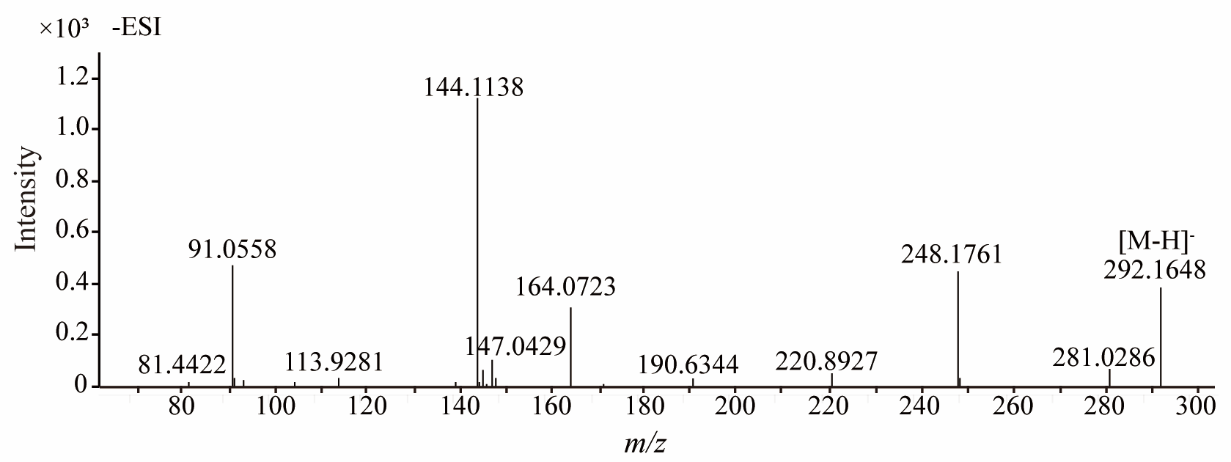


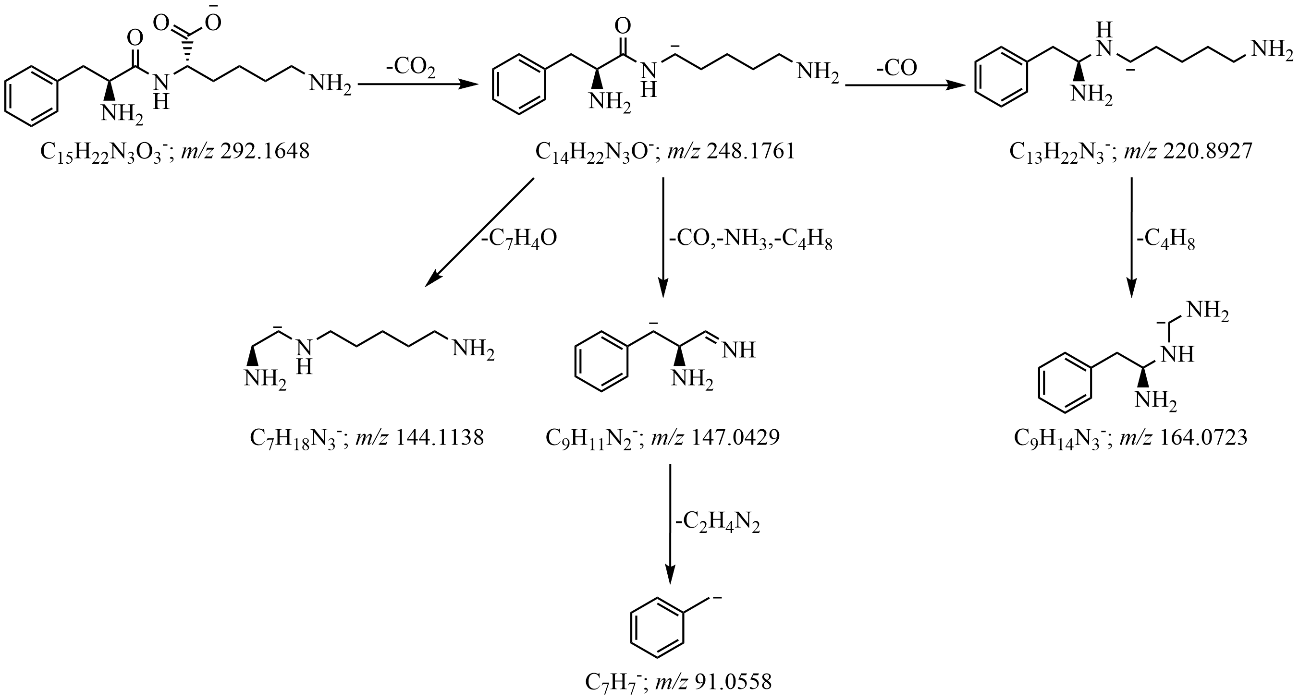


**Figure S10:**The MS/MS spectrum and proposed fragmentation pathways of L-Phe-L-Lys-OH in the negative ion model (compound 6).


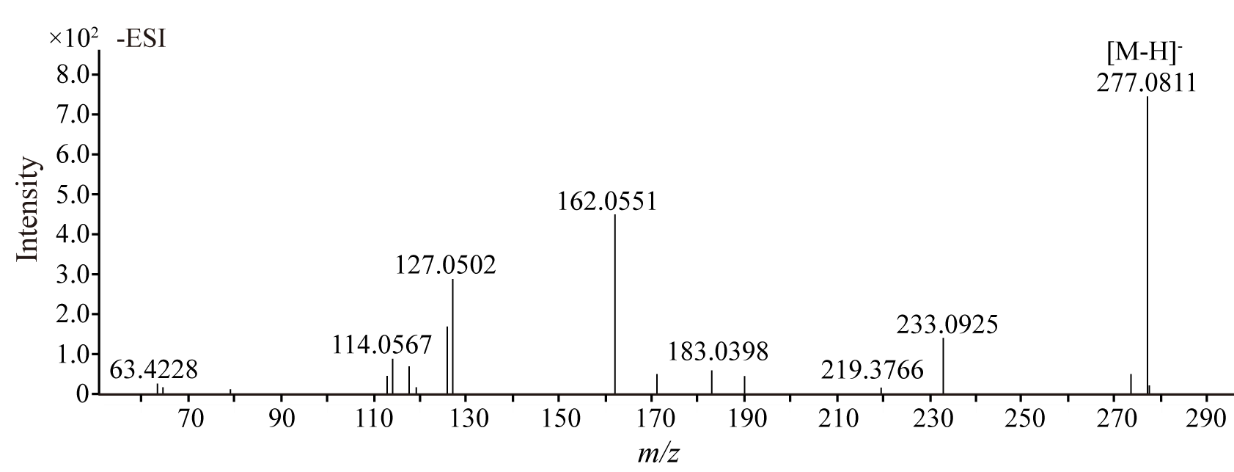


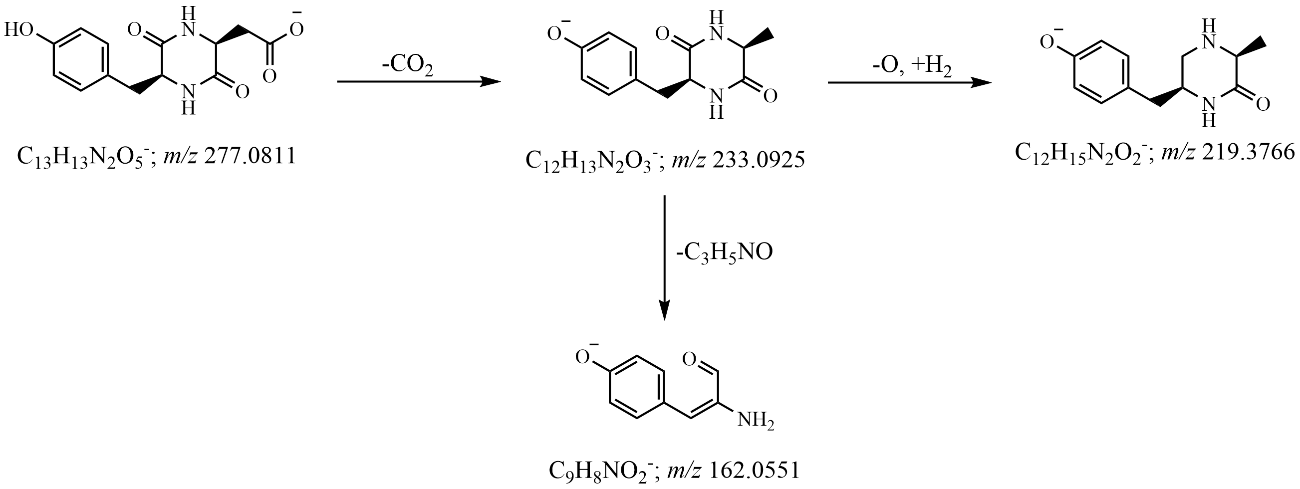


**Figure S11:**The MS/MS spectrum and proposed fragmentation pathways of cyclo(Tyr-Asp) in the negative ion model (compound 23).


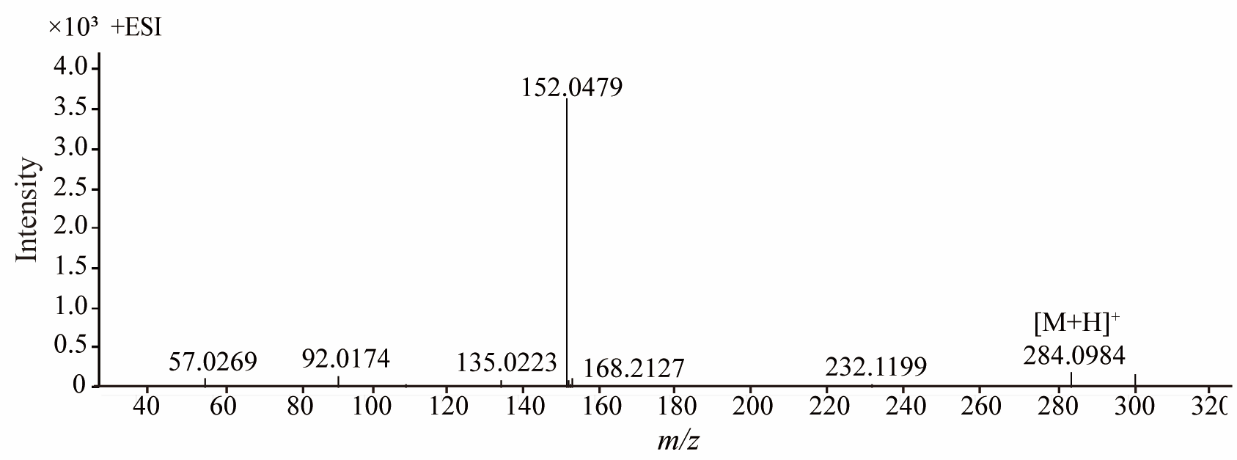


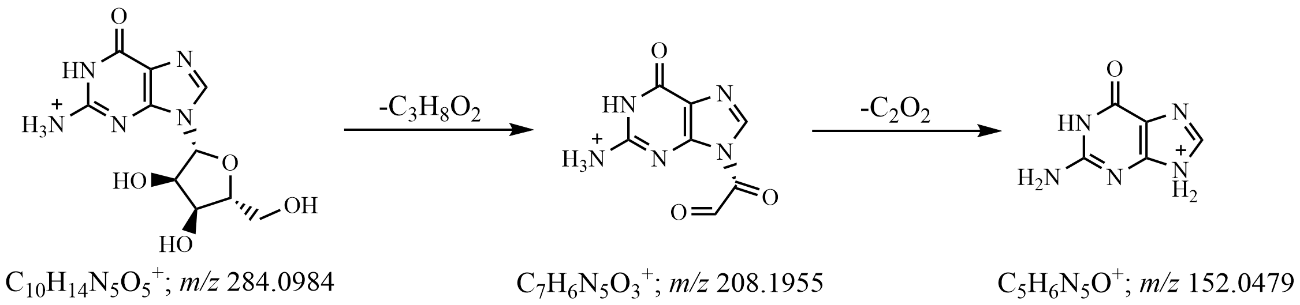


**Figure S12:**The MS/MS spectrum and proposed fragmentation pathways of guanosine in the positive ion model (compound 20).


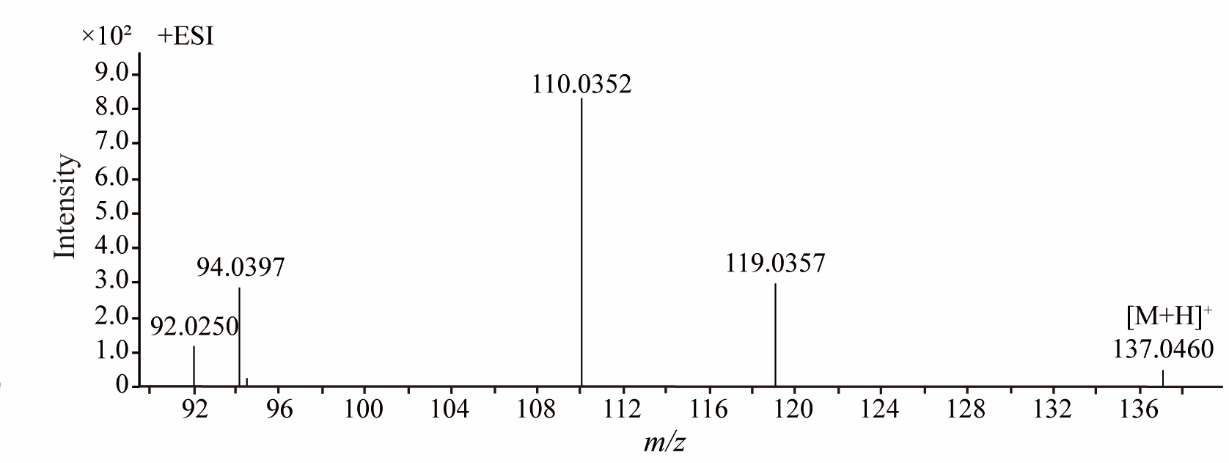


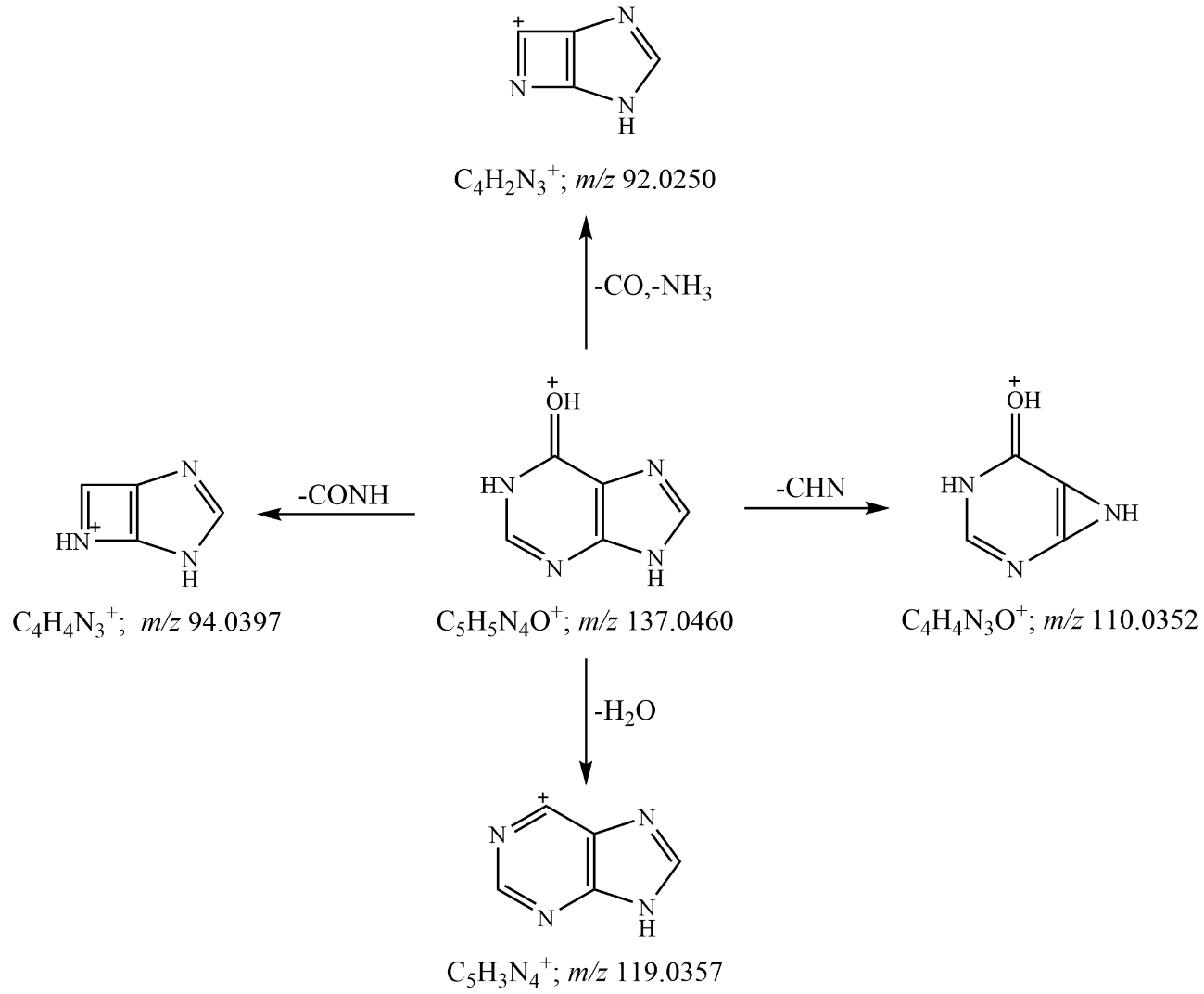


**Figure S13:**The MS/MS spectrum and proposed fragmentation pathways of hypoxanthine in the positive ion model (compound 16).


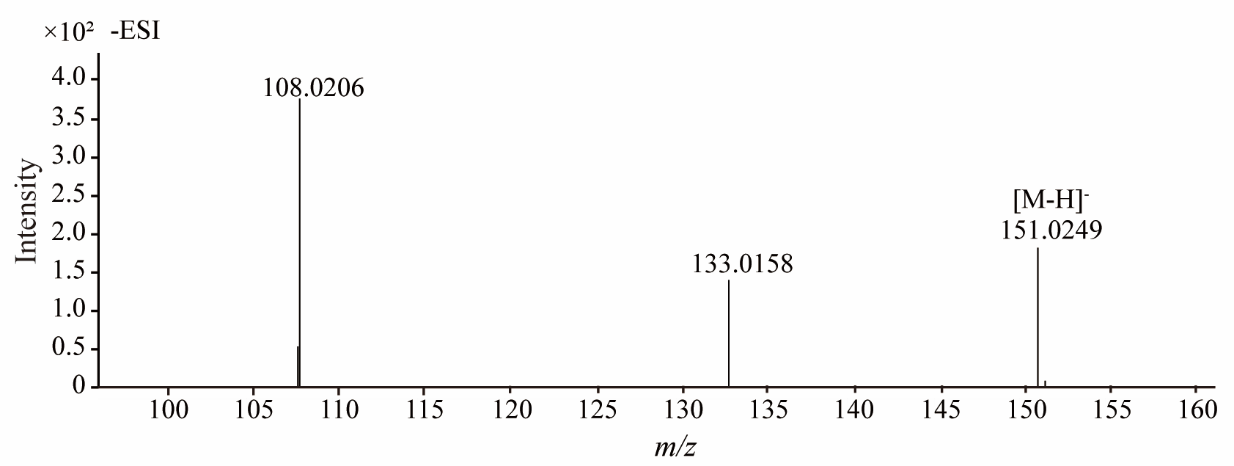


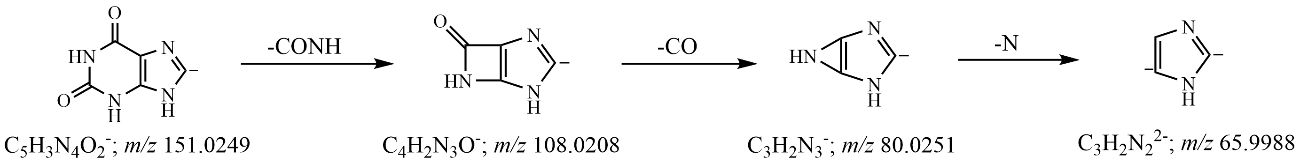


**Figure S14:**The MS/MS spectrum and proposed fragmentation pathways of xanthine in the negative ion model (compound 17).


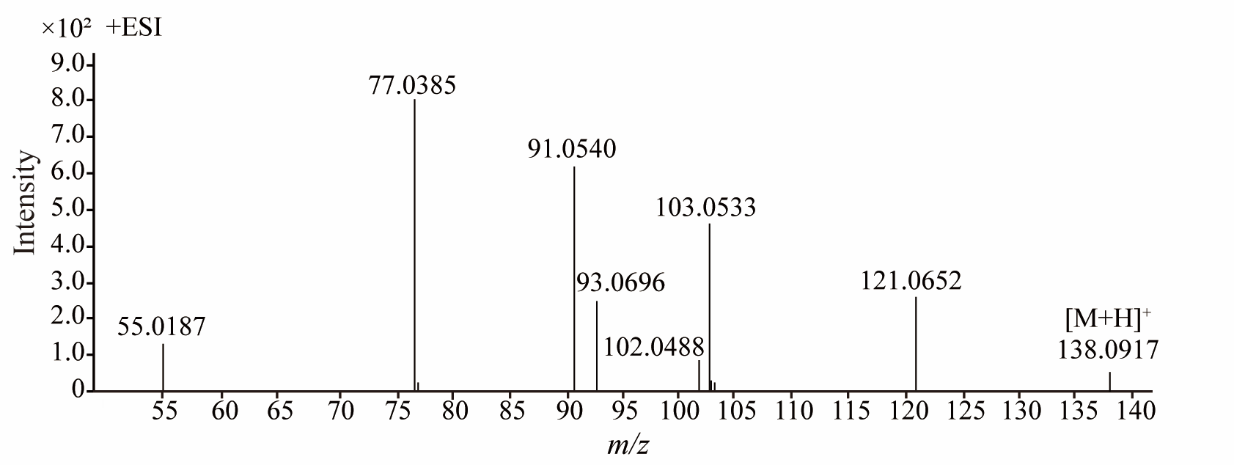


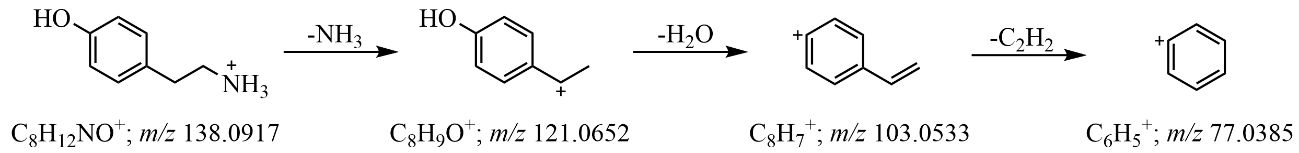


**Figure S15:**The MS/MS spectrum and proposed fragmentation pathways of tyramine in the positive ion model (compound 7).


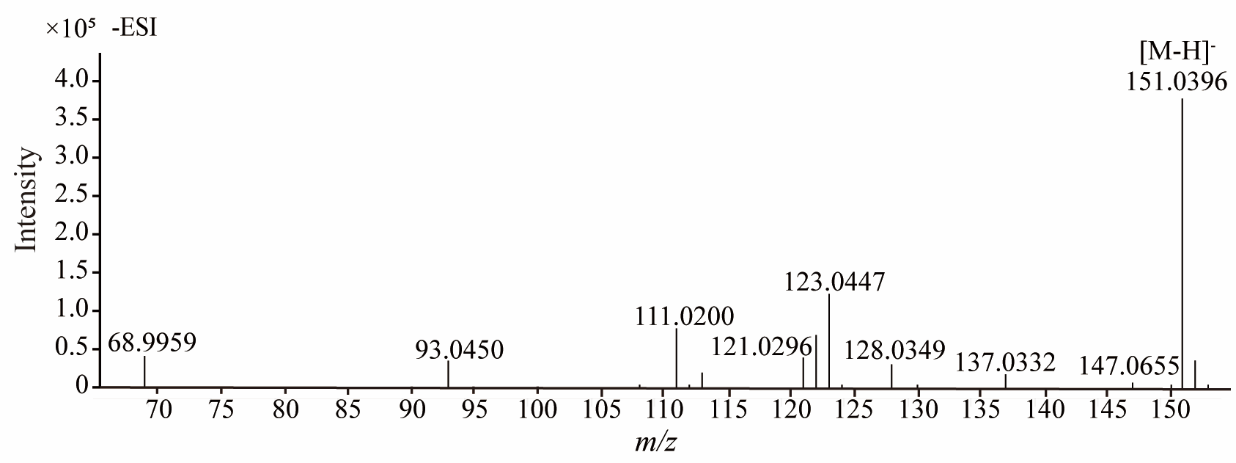


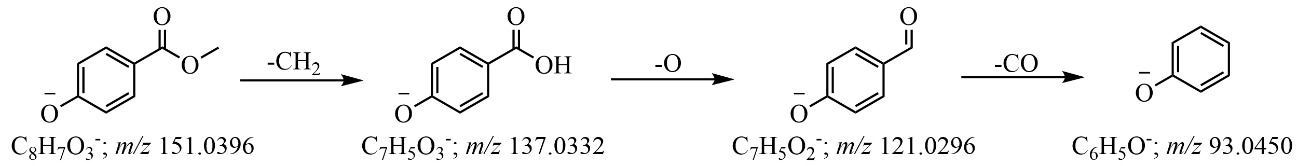


**Figure S16:**The MS/MS spectrum and proposed fragmentation pathways of methyl 4-hydroxybenzoate in the negative ion model (compound 9).


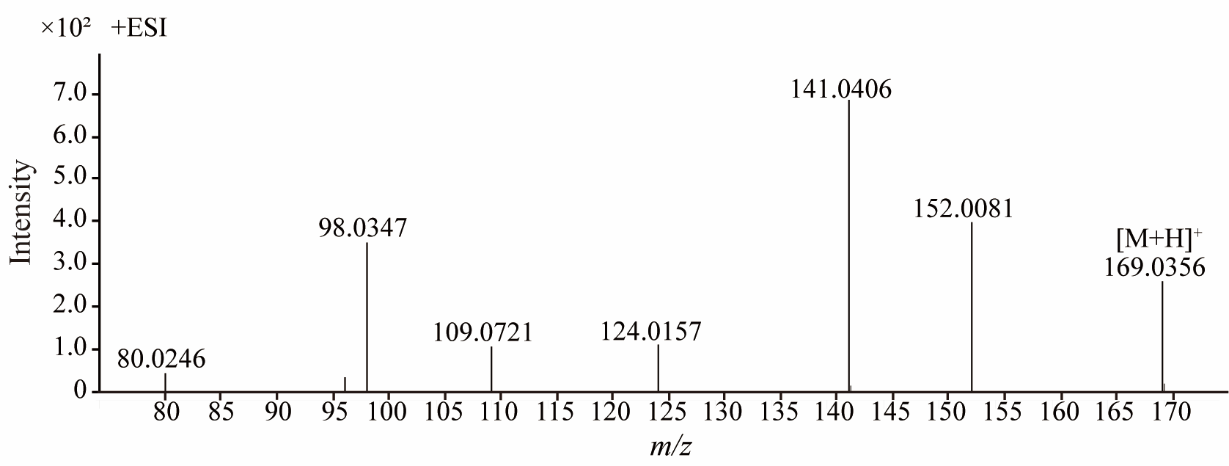


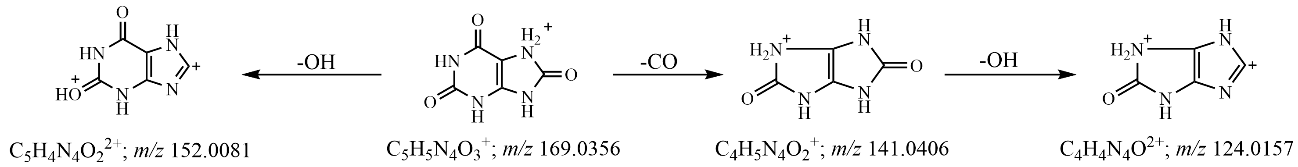


**Figure S17:**The MS/MS spectrum and proposed fragmentation pathways of uric acid in the positive ion model (compound 14).


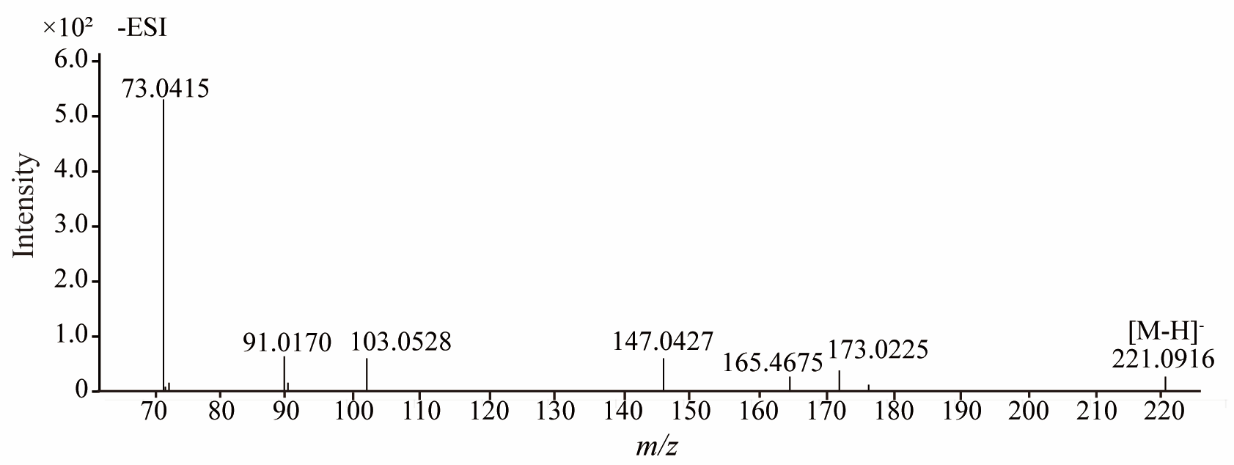


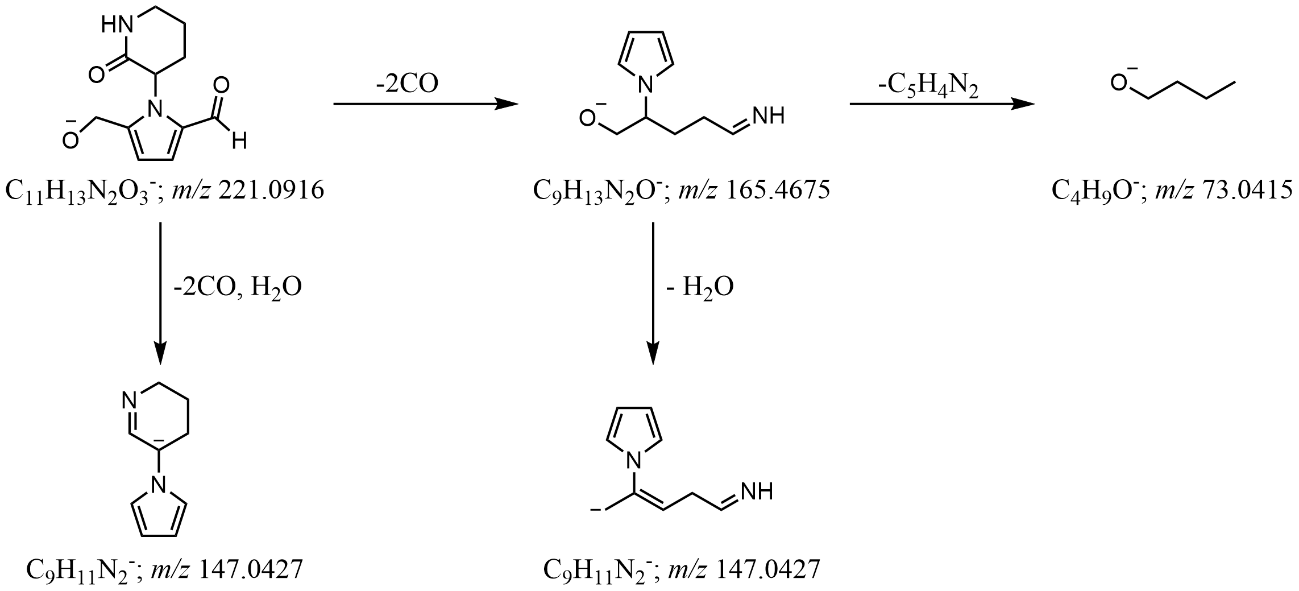


**Figure S18:**The MS/MS spectrum and proposed fragmentation pathways of cordyrrole A in the negative ion model (compound 15).


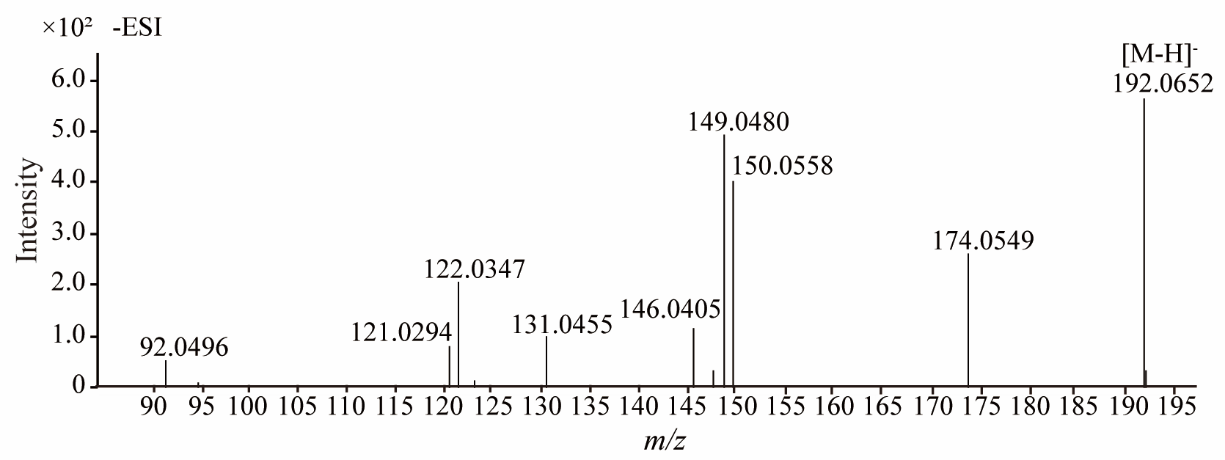


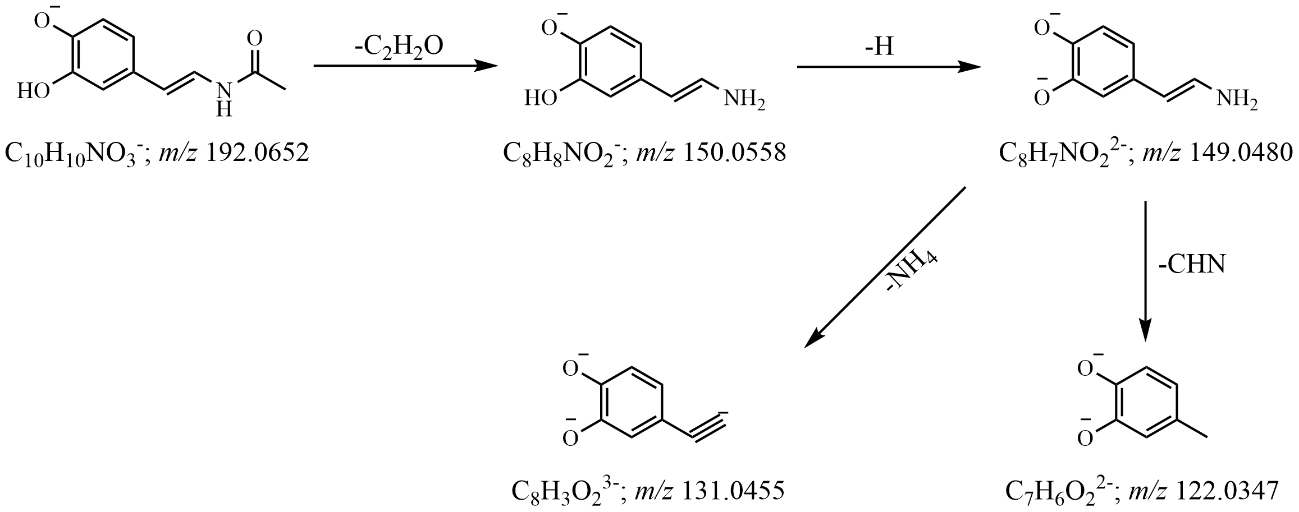


**Figure S19:**The MS/MS spectrum and proposed fragmentation pathways of 1,2-dehydro-N-acetyldopamine in the negative ion model (compound 18).


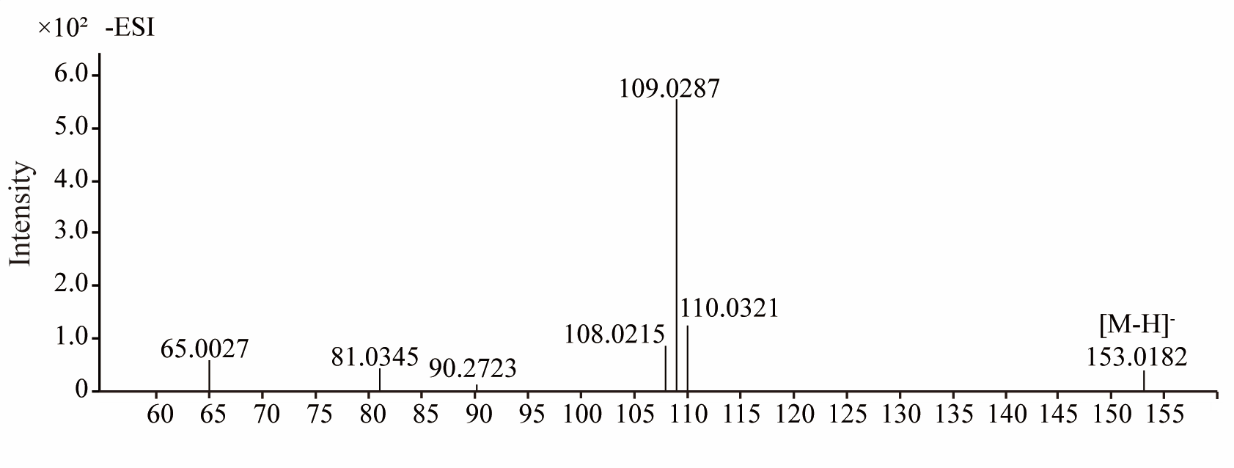


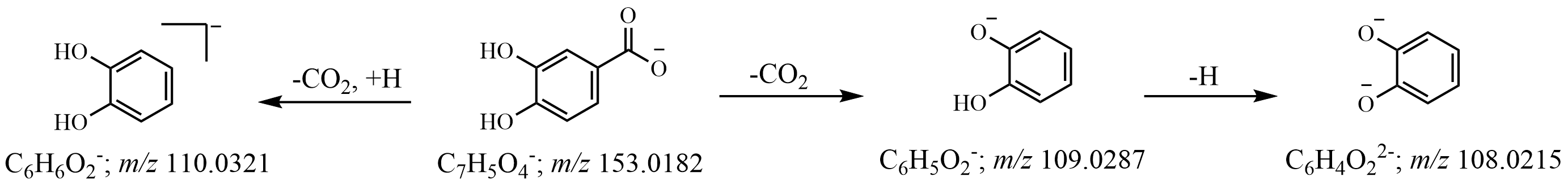


**Figure S20:**The MS/MS spectrum and proposed fragmentation pathways of protocatechuic acid in the negative ion model (compound 24).


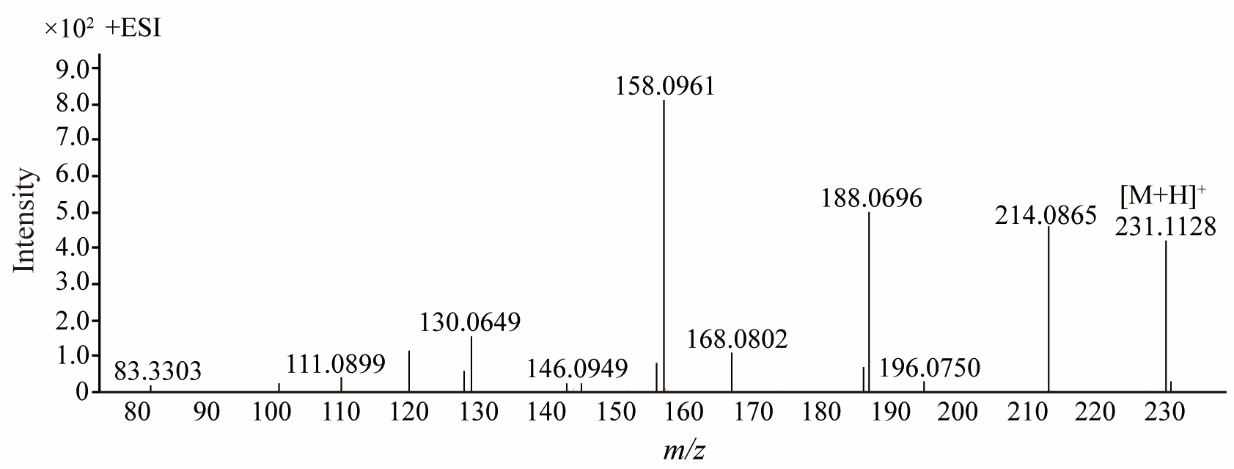


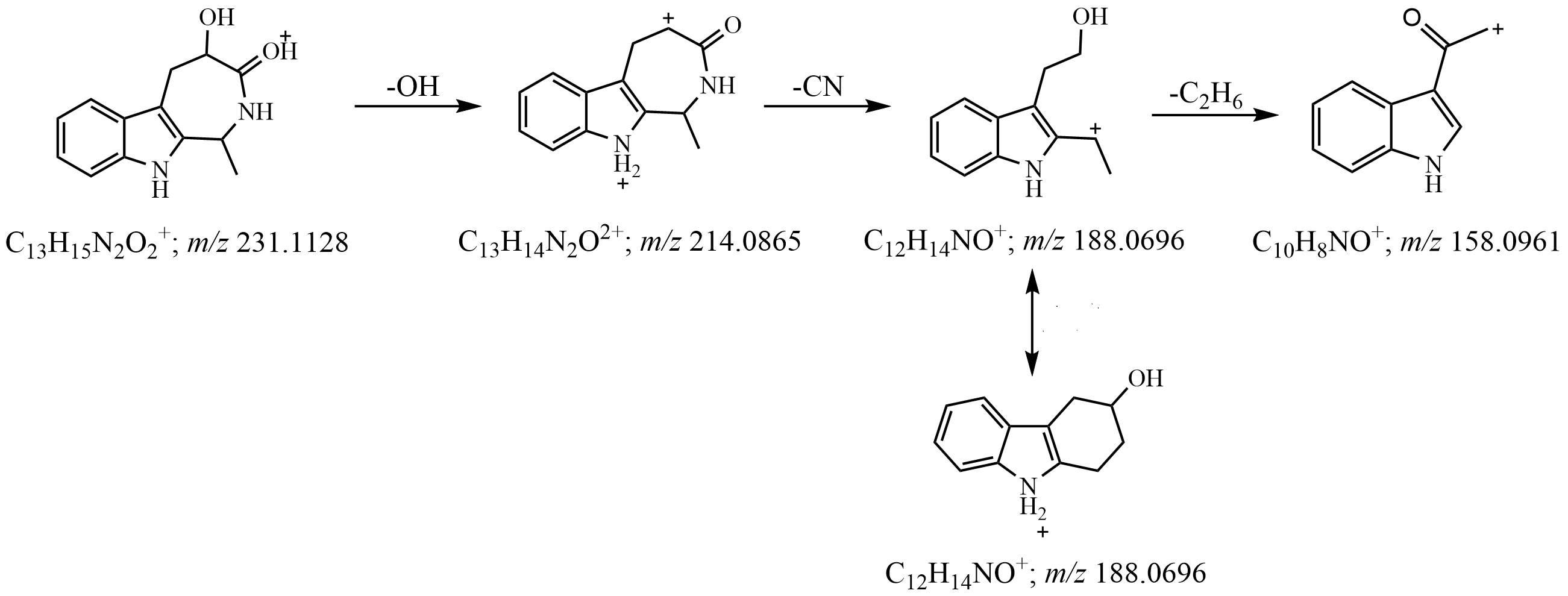


**Figure S21:**The MS/MS spectrum and proposed fragmentation pathways of ginsenine in the positive ion model (compound 25).


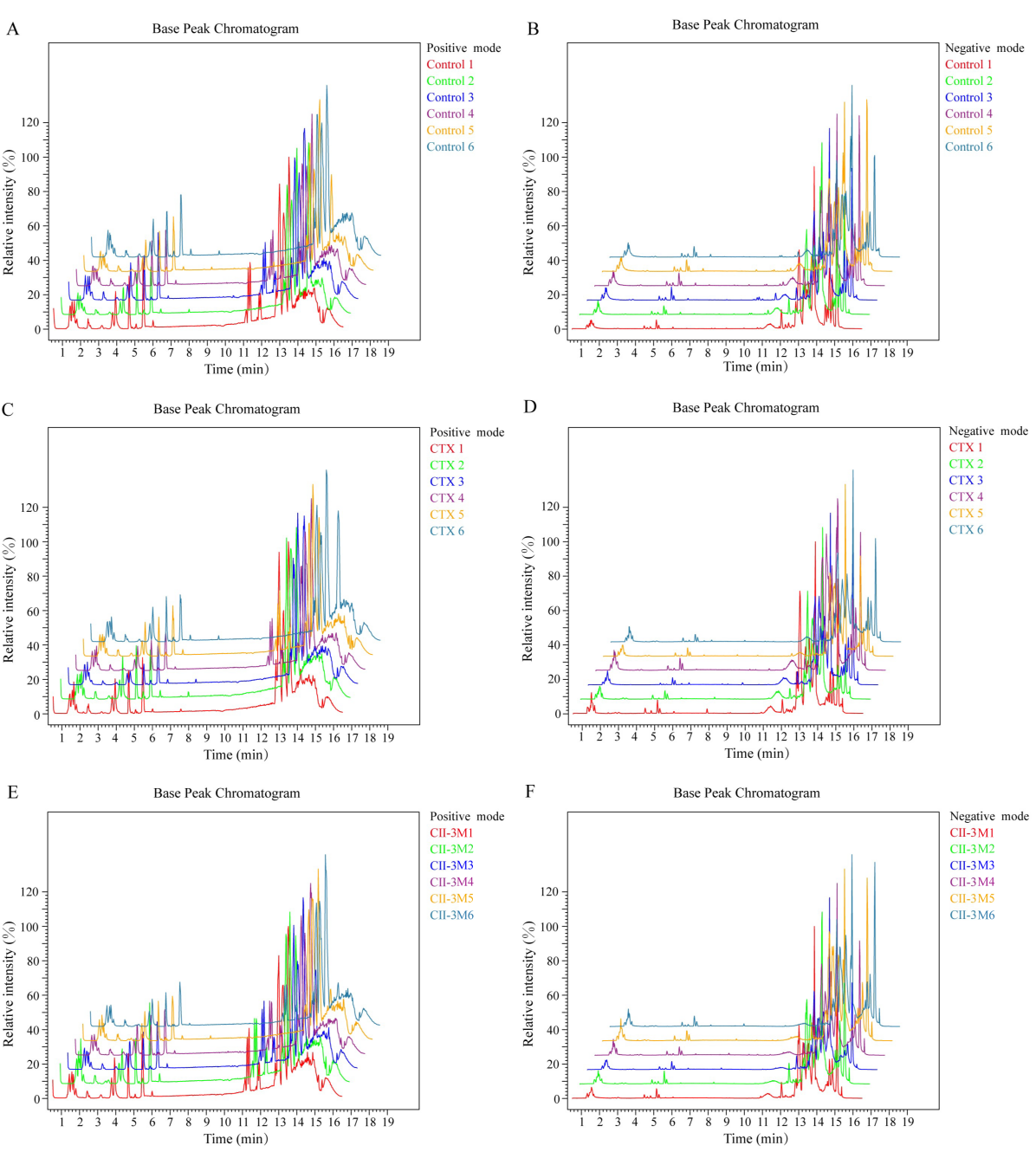


**Figure S22:** BPC of the serum samples detected in positive and negative ion modes.(A) BPC of control group in positive ion mode; (B) BPC of control group in negative ion mode;(C) BPC of CTX group in positive ion mode;(D) BPC of CTX group in negative ion mode;(E) BPC of CⅡ-3M group in positive ion mode; (F) BPC of CⅡ-3M group in negative ion mode.


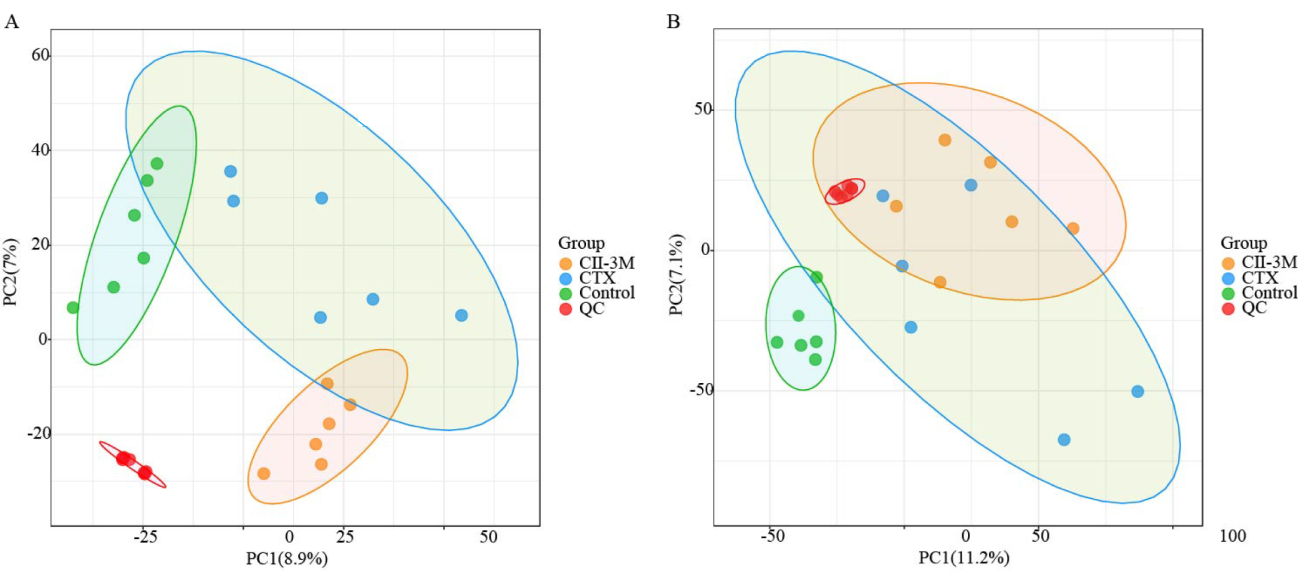


**Figure S23:** PCA score plot for QC samples in positive (A) and negative (B) ion modes.

**Figure S24:** Mirror image of 27 differential metabolites.


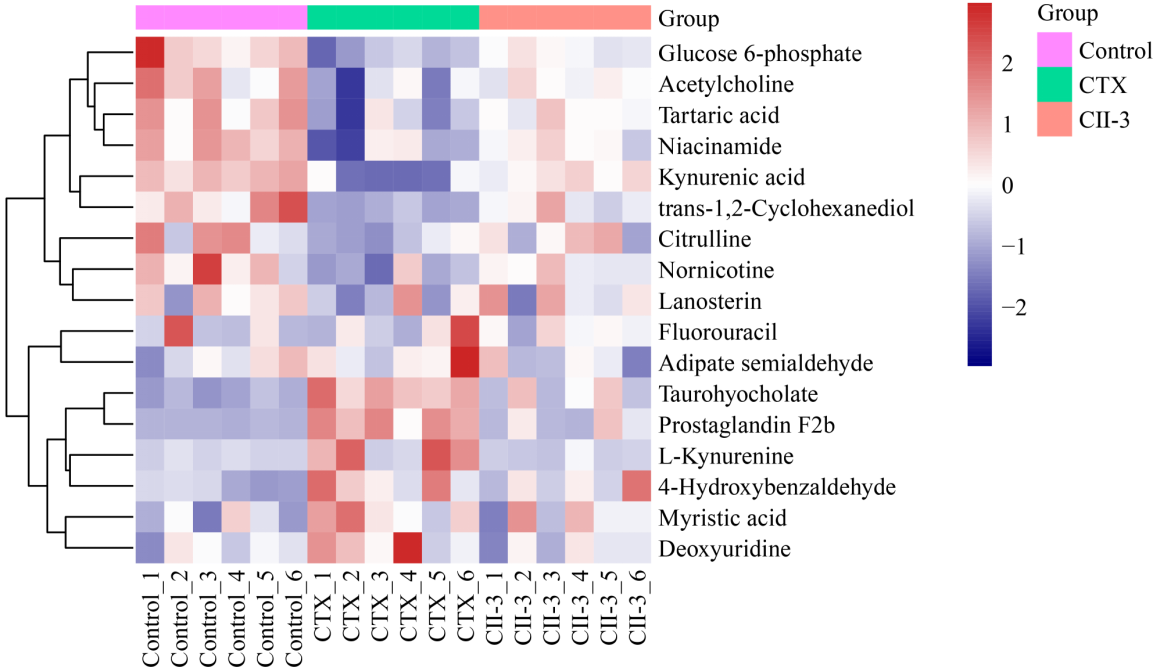


**Figure S25:** Heatmap of differential metabolites identified among the three groups. The red color indicates that the relative content of differential metabolites was high, and the blue color indicates that the relative content of differential metabolites was low.


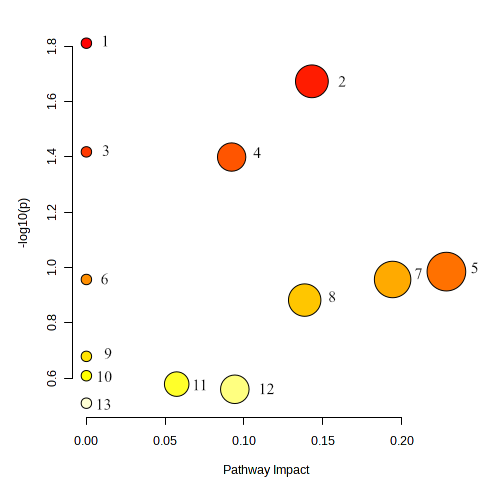


**Figure S26:** Schematic diagram of the potential metabolic pathways in this study. 1:Neomycin, kanamycin and gentamicin biosynthesis; 2: Porphyrin and chlorophyll metabolism; 3: Synthesis and degradation of ketone bodies; 4: Steroid biosynthesis; 5: Arginine biosynthesis; 6: Butanoate metabolism; 7: Nicotinate and nicotinamide metabolism; 8: Starch and sucrose metabolism; 9: Inositol phosphate metabolism; 10: Glycerophospholipid metabolism; 11: Pyrimidine metabolism; 12: Tryptophan metabolism; 13: Fatty acid biosynthesis.


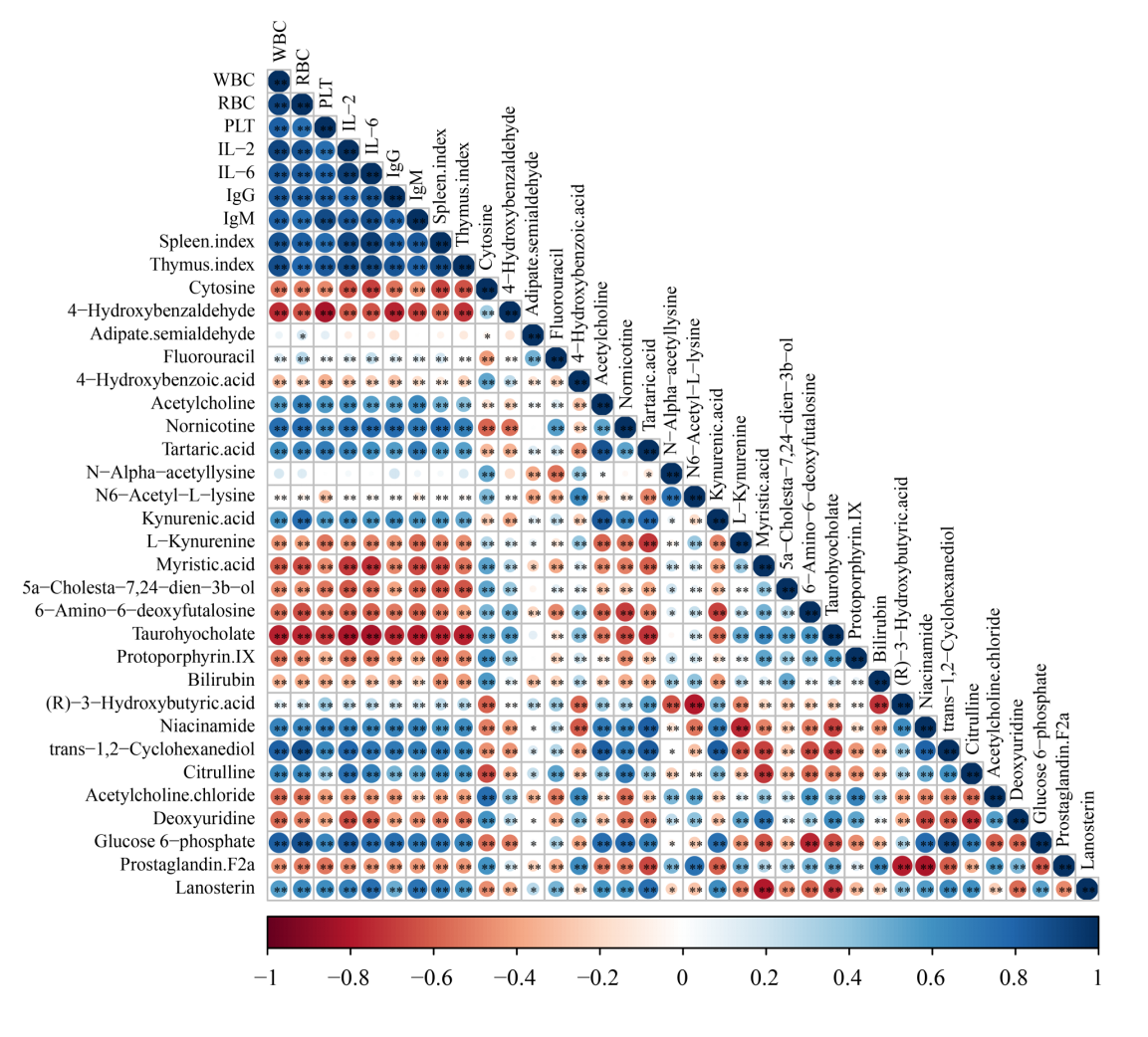


**Figure S27:** Spearman’s correlation analysis between differential metabolites and efficacy indicators among the control, CTX, and CⅡ-3 groups. Red indicates negative correlation, and blue represents positive correlation. **P* < 0.05 and ***P* < 0.01.

Table S2 Identification of compositions in the aqueous extract of CⅡ-3.

| NO. | t_R_/min | Addition ion peak（*m/z*） | | | | | | MS/MS（*m/z*） | Molecular  formula | Identification  Compound | Component  attribution |
| --- | --- | --- | --- | --- | --- | --- | --- | --- | --- | --- | --- |
|  |  | Negative ion (*m*/*z*) | | | Positive ion (*m*/*z*) | | |  |  |  |  |
|  |  | Measured  value | Theoretical value | ppm | Measured  value | Theoretical value | ppm |  |  |  |  |
| 1 | 1.361 | 145.0977 | 145.0977 | 0 | 147.1128 | 147.1134 | -4.1 | 169.0949[M+Na]^+^  147.1128[M+H]^+^  145.0977[M-H]^-^  130.0863[M+H-NH_3_]^+^  84.0808[M+H-NH_2_-COOH-H_2_]^+^  [M+H-NH_3_-COOH-H]^+^ | C_6_H_14_N_2_O_2_ | Lysine* | a |
| 2 | 1.550 | 173.1041 | 173.1039 | 1.2 | 175.1192 | 175.1195 | -1.7 | 523.3418[3M+H]^+^  349.2302[2M+H]^+^  347.8542[2M-H]^-^  175.1192[M+H]^+^  173.1041[M-H]^-^  158.0916[M+H-NH_3_]^+^  130.0963[M+H-NH_3_-CO]^+^  116.0717[M+H-CH_5_N_3_]^+^  112.0873[M+H-NH_3_-COOH-H]^+^  71.0502[M+H-NH_3_-CO-CH_5_N_3_]^+^  70.0753[M+H- CH_5_N_3_-CO-H_2_O]^+^  60.0553[M+H-C_5_H_9_NO_2_]^+^ | C_6_H_14_N_4_O_2_ | Arginine* | a |
| 3 | 1.907 | 258.1808 | 258.1818 | -3.9 | － | － | － | 517.3714[2M-H]^-^  258.1808[M-H]^-^  240.2061[M-H-H_2_O]^-^  213.3517[M-H-H_2_O-CO+H]^-^  166.4611[M-H-H_2_O-C_3_H_6_O_2_]^-^  130.0865[M-H-C_6_H_12_N_2_O]^-^  97.0766[M-H-H_2_O-CO+H-C_6_H_14_NO]^-^ | C_12_H_25_N_3_O_3_ | L-Lys-Leu-OH | b |
| 4 | 2.149 | 286.1866 | 286.1879 | -4.5 | 288.2035 | 288.2036 | -0.3 | 573.3816[2M-H]^-^  286.1866[M-H]^-^  269.1632[M-H-NH_3_]^-^  257.7971[M-H-C_2_H_5_]^-^  244.1660[M-H-C_2_H_5_-CH]^-^  200.1774[M-H-C_2_H_5_-CH-CO_2_]^-^  129.1019[M-H-C_2_H_5_-CH-CO_2_-C_3_H_5_NO]^-^  58.0409[M-H-C_2_H_5_-C_7_H_11_N_4_O_3_]^-^ | C_12_H_25_N_5_O_3_ | L-Leu-Arg-OH | b |
| 5 | 2.450 | 130.0872 | 130.0868 | 3.1 | 132.1019 | 132.1025 | -4.5 | 392.2677[3M-H]^-^  132.1019[M+H]^+^  130.0861[M-H]^-^  86.0965[M+H-NO_2_]^+^ | C_6_H_13_NO_2_ | Leucine | a |
| 6 | 2.702 | 292.1648 | 292.1648 | 0 | － | － | － | 292.1648[M-H]^-^  248.1761[M-H-CO_2_]^-^  220.8927[M-H-CO_2_-CO]^-^  164.0723[M-H-CO_2_-CO-C_4_H_8_]-  147.0429[M-H-CO_2_-CO-NH_3_-C_4_H_8_]^-^  144.1138[M-H-CO_2_-C_7_H_4_O]^-^  91.0558[M-H-CO_2_-CO-NH_3_-C_4_H_8_-C_2_H_4_N_2_]^-^ | C_15_H_23_N_3_O_3_ | L-Phe-L-Lys-OH | b |
| 7 | 3.041 | － | － | － | 138.0917 | 138.0919 | -1.4 | 138.0917[M+H]^+^  121.0652[M+H-NH_3_]^+^  103.0533[M+H-NH_3_-H_2_O]^+^  77.0385[M+H-NH_3_-H_2_O-C_2_H_2_]^+^ | C_8_H_11_NO | Tyramine | e |
| 8 | 3.488 | 180.0653 | 180.0661 | -4.4 | 182.0812 | 182.0817 | -2.7 | 363.1528[2M+H]^+^  182.0812[M+H]^+^  180.0653[M-H]^-^  165.0543[M+H-NH_3_]^+^  147.0413[M+H-NH_3_-H_2_O]^+^  136.0737[M+H-H_2_O-CO]^+^  123.0430[M+H-NH_3_-CH_2_CO]^+^  119.0489[M+H-NH_3_-H_2_O-CO]^+^  91.0532[M+H-NH_3_-H_2_O-2CO]^+^ | C_9_H_11_NO_3_ | Tyrosine* | a |
| 9 | 3.778 | 151.0396 | 151.0395 | 0.7 | － | － | － | 197.0882[M+HCOO]^-^  151.0396[M-H]^-^  137.0332[M-H-CH_2_]^-^  121.0296[M-H-CH_2_-O]^-^  93.0450[M-H-CH_2_-O-CO]^-^ | C_8_H_8_O_3_ | Methyl 4-hydroxybenzoate | e |
| 10 | 3.909 | 178.0868 | 178.0868 | 0.0 | 180.1018 | 180.1025 | -3.9 | 357.2250[2M-H]^-^  180.1018[M+H]^+^  178.0868[M-H]^-^  162.0532[M-H-O]^-^  149.0613[M-H-CHO]^-^  107.2236[M-H-CHO-C_2_H_3_-NH]^-^ | C_10_H_13_NO_2_ | N-Acetyltyramine | e |
| 11 | 4.251 | 164.0718 | 164.0712 | 3.7 | 166.0863 | 166.0868 | -3.0 | 331.1650[2M+H]^+^  166.0863[M+H]^+^  164.0718[M-H]^-^  131.0503[M+H-NH_3_-H_2_O]^+^  120.0810[M+H-H_2_O-CO]^+^  118.0632[M+H-H_2_O-CO-H_2_]^+^  103.0544[M+H -H_2_O-CO-NH_3_]^+^  93.0686[M+H-H_2_O-CO-HCN]^+^  91.0528[M+H-H_2_O-CO-HCN-H_2_]^+^  77.0398[M+H -H_2_O-CO-NH_3_-C_2_H_2_]^+^ | C_9_H_11_NO_2_ | Phenylalanine* | a |
| 12 | 4.675 | 134.0472 | 134.0467 | 3.7 | 136.0618 | 136.0623 | -3.7 | 136.0618[M+H]^+^  134.0472[M-H]^-^  119.0349[M+H-NH_3_]^+^  94.0393[M+H-NH_3_-CN+H]^+^ | C_5_H_5_N_5_ | Adenine* | c |
| 13 | 4.915 | － | － | － | 156.0771 | 156.0773 | -1.3 | 156.0771[M+H]^+^  123.0538[M+H-NH_2_-OH]^+^  110.0699[M+H-CO-H_2_O]^+^ | C_6_H_9_N_3_O_2_ | Histidine | a |
| 14 | 5.454 | 167.0211 | 167.0205 | 3.4 | 169.0356 | 169.0362 | -3.5 | 335.0494[2M-H]^-^  213.1240[M+HCOO]^-^  169.0356[M+H]^+^  152.0081[M+H-OH]^+^  141.0406[M+H-CO]^+^  124.0157[M+H-CO-OH]^+^ | C_5_H_4_N_4_O_3_ | Uric acid | e |
| 15 | 6.468 | 221.0916 | 221.0926 | -4.5 | － | － | － | 443.1943[2M-H]^-^  221.0916[M-H]^-^  165.4675[M-H-2CO]^-^  147.0427[M-H-2CO-H_2_O]^-^  73.0415[M-H-2CO-C_5_H_4_N_2_]^-^ | C_11_H_14_N_2_O_3_ | Cordyrrole A | e |
| 16 | 6.699 | 135.0312 | 135.0307 | 3.7 | 137.0460 | 137.0463 | -2.2 | 295.0649[2M+Na]^+^  273.0830[2M+H]^+^  159.0465[M+Na]^+^  137.0460[M+H]^+^  135.0312[M-H]^-^  119.0357[M+H-H_2_O]^+^  110.0352[M+H-CHN]^+^  94.0397[M+H-CONH]^+^  92.0250[M+H-CO-NH_3_]^+^ | C_5_H_4_N_4_O | Hypoxanthine* | c |
| 17 | 7.647 | 151.0249 | 151.0256 | -4.6 | － | － | － | 175.0852[M+Na]^+^  153.0358[M+H]^+^  151.0249[M-H]^-^  108.0208[M-H-CONH]^-^  80.0251[M-H-CONH-CO]^-^  65.9988[M-H-CONH-CO-N]^2-^ | C_5_H_4_N_4_O_2_ | Xanthine* | c |
| 18 | 7.919 | 192.0652 | 192.0661 | -4.7 | － | － | － | 194.0798[M+H]^+^  192.0652[M-H]^-^  150.0558[M-H-C_2_H_2_O]^-^  149.0480[M-H-C_2_H_2_O-H]  131.0455[M-H-C_2_H_2_O-H-NH_4_]^-^  122.0347[M-H-C_2_H_2_O-H-CHN]^-^ | C_10_H_11_NO_3_ | 1,2-dehydro-N-acetyldopamine | e |
| 19 | 8.331 | 267.0720 | 267.0729 | -3.4 | 269.0884 | 269.0886 | -0.7 | 537.1681[2M+H]^+^  291.0689[M+Na]^+^  269.0884[M+H]^+^  267.0720[M-H]^-^  209.1286[M+H-C_2_H_4_O_2_]^+^  137.0440[M+H-C_5_H_8_O_4_]^+^ | C_10_H_12_N_4_O_5_ | Inosine* | d |
| 20 | 8.806 | 282.0829 | 282.0838 | -3.2 | 284.0984 | 284.0995 | -3.9 | 567.1900[2M+H]^+^  306.0797[M+Na]^+^  284.0984[M+H]^+^  282.0829[M-H]^-^  208.1955[M+H-C_7_H_8_O_2_]^+^  152.0479[M+H-C_2_O_2_]^+^ | C_10_H_13_N_5_O_5_ | Guanosine* | d |
| 21 | 8.903 | 203.0829 | 203.0821 | 3.9 | 205.0972 | 205.0977 | -2.4 | 409.1871[2M+H]^+^  205.0972[M+H]^+^  203.0829[M-H]^-^  188.0697[M+H-NH_3_]^+^  170.0582[M+H-NH_3_-H_2_O]^+^  159.0903[M+H-H_2_O-CO]^+^  146.0587[M+H-NH_3_-CH_2_CO]^+^  132.0793[M+H-H_2_O-CO-HCN]^+^  130.0634[M+H- H_2_O-CO-HCN-H_2_]^+^  118.0641[M+H-NH_3_CH_2_CO-CO]^+^  91.0540[M+H- NH_3_-CH_2_CO-CO-HCN]^+^  74.0230[M+H-C_9_H_9_N]^+^ | C_11_H_12_N_2_O_2_ | Tryptophan* | a |
| 22 | 11.001 | － | － | － | 169.0972 | 169.0977 | -3.0 | 337.1872[2M+H]^+^  191.0779[M+Na]^+^  169.0972[M+H]^+^  152.0675[M+H-OH]^+^  89.9151[M+H-C_5_H_5_N]^+^  72.0432[M+H-C_4_H_3_NO_2_]^+^  70.0650[M+H-C_4_H_3_NO_2_-H_2_]^+^ | C_8_H_12_N_2_O_2_ | cyclo(Ala-Pro) | b |
| 23 | 12.047 | 277.0811 | 277.0824 | -4.7 | － | － | － | 555.3439[2M-H]^-^  277.0811[M-H]^-^  233.0925[M-H-CO_2_]^-^  219.3766[M-H-CO_2_-O+H_2_]^-^  162.0551[M-H-CO_2_-C_3_H_5_NO]^-^ | C_13_H_14_N_2_O_5_ | cyclo(Tyr-Asp) | b |
| 24 | 12.494 | 153.0182 | 153.0188 | -3.9 | － | － | － | 307.0792[2M-H]^-^  199.0586[M+HCOO]^-^  153.0182[M-H]^-^  110.0321[M-H-CO_2_+H]^-^  109.0287[M-H-CO_2_]^-^  108.0215[M-H-CO_2_-H]^-^ | C_7_H_6_O_4_ | Protocatechuic acid | e |
| 25 | 15.729 | 229.0968 | 229.0977 | -3.9 | 231.1128 | 231.1134 | -2.6 | 253.1680[M+Na]^+^  231.1128[M+H]^+^  229.0968[M-H]^-^  214.0865[M+H-OH]^+^  188.0696[M+H-OH-CN]^+^  158.0961[M+H-OH-CN-C_2_H_6_]^+^ | C_13_H_14_N_2_O_2_ | Ginsenine | e |

Notes: a: Amino acids; b: Dipeptides and cyclic peptides; c: Purines; d: Nucleosides; e: Other compounds. *: Compared with reference standards.

Table S3. The RSDs of the retention time and peak area of 10 extracted ions in QC samples.

| *m/z* | Rt（s） | Rt (RSD %) | Area | Area (RSD %) |
| --- | --- | --- | --- | --- |
| 301.1727(ESI^+^) | 96.35±0.56 | 0.59 | 7780090.54±94164.38 | 1.21 |
| 104.0426(ESI^-^) | 98.17±0.26 | 0.26 | 25835719.36±512219.41 | 1.98 |
| 112.0503(ESI^+^) | 147.11±1.05 | 0.71 | 149780351.90±4126110.07 | 2.75 |
| 132.0660(ESI^+^) | 249.34±0.33 | 0.13 | 8269815.37±168754.12 | 2.04 |
| 146.9368(ESI^-^) | 260.69±2.97 | 1.14 | 4837369.03±108779.66 | 2.25 |
| 100.1123(ESI^+^) | 646.49±1.94 | 0.30 | 732812883.38±78182195.60 | 10.67 |
| 110.0201(ESI^+^) | 733.22±3.68 | 0.50 | 95073116.26±10699299.41 | 11.25 |
| 481.2347(ESI^-^) | 748.08±0.50 | 0.07 | 7136866.42±263174.07 | 3.69 |
| 483.2700(ESI^-^) | 759.95±0.78 | 0.10 | 1887899.89±35844.59 | 1.90 |
| 115.9187(ESI^-^) | 856.16±1.28 | 0.15 | 121363862.75±6566604.64 | 5.41 |
